# Cadmium exposure and sulfate limitation reveal differences in the transcriptional control of three sulfate transporter (*Sultr1;2*) genes in *Brassica juncea*

**DOI:** 10.1186/1471-2229-14-132

**Published:** 2014-05-16

**Authors:** Clarissa Lancilli, Barbara Giacomini, Giorgio Lucchini, Jean-Claude Davidian, Maurizio Cocucci, Gian Attilio Sacchi, Fabio Francesco Nocito

**Affiliations:** 1 Dipartimento di Scienze Agrarie e Ambientali – Produzione, Territorio, Agroenergia, Università degli Studi di Milano, 20133 Milano, Italy; 2 Biochimie et Physiologie Moléculaire des Plantes, Unité mixte de recherche, Montpellier SupAgro (Département Biologie et Ecologie), INRA, CNRS, Université de Montpellier 2, 34060 Montpelliercedex 2, France

**Keywords:** *Brassica juncea*, Cadmium, Sulfate limitation, High-affinity sulfate transporters

## Abstract

**Background:**

Cadmium (Cd) exposure and sulfate limitation induce root sulfate uptake to meet the metabolic demand for reduced sulfur. Although these responses are well studied, some aspects are still an object of debate, since little is known about the molecular mechanisms by which changes in sulfate availability and sulfur metabolic demand are perceived and transduced into changes in the expression of the high-affinity sulfate transporters of the roots. The analysis of the natural variation occurring in species with complex and highly redundant genome could provide precious information to better understand the topic, because of the possible retention of mutations in the sulfate transporter genes.

**Results:**

The analysis of plant sulfur nutritional status and root sulfate uptake performed on plants of *Brassica juncea* – a naturally occurring allotetraploid species – grown either under Cd exposure or sulfate limitation showed that both these conditions increased root sulfate uptake capacity but they caused quite dissimilar nutritional states, as indicated by changes in the levels of nonprotein thiols, glutathione and sulfate of both roots and shoots. Such behaviors were related to the general accumulation of the transcripts of the transporters involved in root sulfate uptake (*BjSultr1;1* and *BjSultr1;2*). However, a deeper analysis of the expression patterns of three redundant, fully functional, and simultaneously expressed Sultr1;2 forms (*BjSultr1;2a*, *BjSultr1;2b*, *BjSultr1;2c*) revealed that sulfate limitation induced the expression of all the variants, whilst *BjSultr1;2b* and *BjSultr1;2c* only seemed to have the capacity to respond to Cd.

**Conclusions:**

A novel method to estimate the apparent k_M_ for sulfate, avoiding the use of radiotracers, revealed that BjSultr1;1 and BjSultr1;2a/b/c are fully functional high-affinity sulfate transporters. The different behavior of the three *BjSultr1;2* variants following Cd exposure or sulfate limitation suggests the existence of at least two distinct signal transduction pathways controlling root sulfate uptake in dissimilar nutritional and metabolic states.

## Background

Sulfur is an essential element for all living organisms, since it is found in a broad variety of biological compounds playing pivotal roles in a number of metabolic processes [1]. In contrast to animals, which have a dietary requirement for some organic sulfur compounds, plants have metabolic pathways that allow them to assimilate inorganic sulfur into organic sulfur compounds through a cascade of well characterized enzymatic steps. For this reason plant sulfur assimilatory pathways are considered to be the main sources of organic sulfur compounds for animal and human diets [2].

The main sulfur source for plants is the sulfate ion of the soil solution available in the rhizosphere [3,4], which is taken up through specific root plasma membrane high-affinity sulfate transporters. Once inside the plant, sulfate is allocated to different sinks, and undergoes intracellular channeling to chloroplast and vacuole, where it is assimilated into organic sulfur compounds or compartmentalized as sulfur store, respectively [2]. The main pathway of sulfate assimilation in plants involves the adenylation of the anion and its stepwise reduction to sulfite and then sulfide which is finally incorporated via *O*-acetylserine (OAS) into cysteine (Cys), a key intermediate from which the essential amino acid methionine (Met), the tripeptide glutathione (GSH), and most sulfur containing compounds are synthesized [2,5].

Considering the central role of Cys in sulfur metabolism, it appears evident that both sulfate uptake and the reductive assimilation pathway have to be finely modulated to meet the metabolic demand for sulfur arising from Cys consuming activities, which largely contribute to define the total sulfur requirement of plants. Such a demand may consistently vary under the different environmental conditions that plants may experience during their growth. For instance, biotic and abiotic stresses may increase the metabolic demand for some Cys derived compounds, causing an increase in the activity of the sulfate assimilatory pathway [6]. An example of this has been largely described in plants exposed to cadmium (Cd) in which the activation of a wide range of adaptive responses involving GSH consuming activities may increase the demand for sulfate, sulfur metabolites and carbon skeletons [7-10]. Indeed, GSH not only acts as an antioxidant in mitigating Cd-induced oxidative stress, but also represents the key intermediate for the synthesis of phytochelatins (PCs), a class of Cys-rich heavy metal-binding peptides involved in buffering cytosolic metal-ion concentration [11]. The large amount of PCs produced by Cd stressed plants represents an additional sink for reduced sulfur which, by increasing the metabolic request for both Cys and GSH, generates a typical demand-driven coordinated transcriptional regulation of genes involved in sulfate uptake, sulfate assimilation and GSH biosynthesis. Such a response is thought to be essential to satisfy two contrasting needs arising from Cd stress: i) maintaining cell GSH homeostasis; ii) detoxifying heavy metals by means of GSH-consuming activities. A similar activation has been described under sulfate limitation [12-14], although in this condition plant sulfur needs to sustain the growth do not vary: the induction of sulfate transporters and enzymes along the assimilatory pathway reflects some difficulties in maintaining both an adequate rate of Cys biosynthesis and sulfur-containing compound homeostasis.

Sulfate transport activations under Cd stress and sulfate limitation have been shown to be mainly controlled at transcriptional level and have been often indicated as resulting from the same, although controversial, nutritional signals [8,9,15]. In the current model of transcriptional regulation, some intermediates along the pathway of sulfate assimilation and GSH biosynthesis act as negative or positive signals in modulating the expression of sulfate transporters. Adequate levels of reduced sulfur compounds, such as Cys and GSH, would repress gene expression through a negative feedback loop preventing excessive sulfate uptake and reduction; *vice versa* a contraction of GSH pools would de-repress gene transcription allowing sulfate to enter the pathway. A second regulatory loop, involving OAS as a key intermediate, should act in promoting gene de-repression when nitrogen and carbon supply exceeds sulfur availability within the cells. In this condition, since sulfide availability is not enough for Cys biosynthesis, OAS accumulates and partially overrides the negative feedback provided by GSH on gene transcription [16]. Such a reversible regulation allows the system to adjust sulfate uptake to the nutritional status of the plant, and agrees with the concept of demand-driven regulation of sulfate uptake and metabolism [12].

Comparative studies clearly show that both sulfate deprivation and Cd stress produce a contraction in the GSH pools and a positive change in the OAS levels, which in turn may induce the accumulation of high-affinity sulfate transporter mRNAs, allowing sulfate to enter the cells [15]. However, some aspects of this picture need to be further investigated, since the relationships existing between the accumulation of sulfate transporter mRNAs and the levels of the signal-intermediates do not always appear to be evident [9,17]. Moreover, Rouached and co-workers [15] clearly showed that the expression of the Arabidopsis *Sultr1;1* and *Sultr1;2* – two high-affinity sulfate transporter genes – is not regulated in complete agreement with the current model, and they proposed the existence of distinct signaling pathways controlling sulfate uptake under different sulfur nutritional status. Finally, whether cellular contents of sulfate, sulfide, OAS, Cys and GSH are the true primary signals for controlling sulfate uptake and reduction or rather act indirectly is still a matter of investigation [14,18], since very little is known about the molecular mechanisms involved in the nutritional signal perception and transduction [2,19,20]. Thus the need for additional efforts and integrated experimental approaches appears particularly evident to unveil this picture. The analysis of the natural variation occurring in species with redundant genomes could provide precious information about the molecular mechanisms controlling sulfate uptake, since the presence of redundant genes may have led to the accumulation of mutations which otherwise would have been eliminated by natural selection. From this point of view the species belonging to the *Brassica* genus could be very useful, since several lines of evidence suggest that the genomes of the three diploid *Brassica* species (*B. rapa*, *B. oleracea* and *B. nigra*) are composed of three rearranged variants of an ancestral genome – structurally similar to that of *Arabidopsis thaliana* – and descended from a common mesohexaploid ancestor [21-23]. Moreover the level of complexity may be further increased by considering the allopolyploid *Brassica* species in which two distinct *Brassica* genomes cohabit [24], increasing the probability of evolving novel gene interactions through the processes of sub-functionalization and/or neo-functionalization of paralogs [25,26].

In this work we present and discuss some evidence toward the existence of multiple transduction pathways controlling sulfate uptake under Cd stress and sulfate limitation in *Brassica juncea* (AABB, *n* = 18), a natural occurring allotetraploid species formed through hybridization between *B. rapa* (AA, *n* = 10) and *B. nigra* (BB, *n* = 8), as described by the “triangle of U” [24].

## Methods

### Plant material, growth conditions, and experimental design

*Brassica juncea* L. Czern & Coss (Lodi selection) seeds were sown on filter paper saturated with distilled water and incubated at 26°C in the dark. Three days after sowing, seedlings selected for uniform growth were transplanted into 5 L plastic tanks (6 seedlings per tank) containing an aerated complete nutrient solution [500 μM NH_4_H_2_PO_4_, 3 mM KNO_3_, 2 mM Ca(NO_3_)_2_, 1 mM MgSO_4_, 25 μM Fe-tartrate, 46 μM H_3_BO_3_, 9 μM MnCl_2_, 0.8 μM ZnCl_2_, 0.3 μM CuCl_2_, 0.1 μM (NH_4_)_6_Mo_7_O_24_, pH 6.5] and kept for 14 days (pre-growing period) in a growth chamber maintained at 26°C and 80% relative humidity, with a 16-h light period. For Cd treatments, plants were grown for an additional 8 days (acclimation period) in a 5-fold diluted (not for micronutrients) nutrient solution (acclimation solution) and then exposed to different Cd concentrations (0, 10, and 25 μM CdCl_2_) for 48 h. For sulfate limitation treatments, at the end of the pre-growing period plants were grown for 10 days in the acclimation solution containing different sulfate concentrations (200, 50 or 10 μM); in the cases of the lowest sulfate concentrations, MgCl_2_ was added to maintain the same concentration of magnesium. In both cases the growth chamber parameters were the same as described before, and all hydroponic solutions were renewed twice a week to minimize nutrient depletion. At the end of the experimental periods, plants were immediately used for the *in vivo* experiments or harvested to be further analyzed. In this case roots were washed for 10 min in ice-cold 5 mM CaCl_2_ solution to displace extracellular Cd [27], rinsed in distilled water and gently blotted with paper towels; shoots were separated from roots and the tissues were frozen in liquid N_2_ and stored at -80°C.

### RNA extraction and cDNA cloning

*BjSultr1;1* and *BjSultr1;2* partial cDNAs were amplified by RT-PCR from *Brassica juncea* mRNA isolated from roots. Total RNA was extracted from roots of sulfur-starved plants using TRIzol reagent (LifeTechnologies), poly A^+^ mRNA was isolated using the Oligotex mRNA Spin-Column system (QIAGEN), and first-strand cDNA synthesis was carried out using the SuperScriptIII first-strand synthesis system for RT-PCR (LifeTechnologies) according to the manufacturer's instructions. Degenerate primers BjSultr1;1_degdir_ (5'-ACGGAGGAGGGTCCGRTGCAA-3'), BjSultr1;1_degrev_ (5'-TTYGGGTCGATCACGGCCTGGCA-3'), BjSultr1;2_degdir_ (5'-GTYTTCGATTGGGGRCGTAR-3'), and BjSultr1;2_degrev_ (5'-RAGGAAGAGCAATGTCAAGAGA-3'), were designed based on highly conserved regions identified in sequences of sulfate transporter cDNAs of *Brassica napus* and *Arabidopsis thaliana* [for *BjSultr1;1*: *BnSultr1;1* (GenBank accession no. AJ416460) and *AtSultr1;1* (TAIR accession no. At4g08620); for *BjSultr1;2*: *BnSultr1;2* (GenBank accession no. AJ311388), and *AtSultr1;2* (TAIR accession no. At1g78000)]. 5′- and 3′-regions of the sulfate transporter cDNAs were isolated by 5′- and 3′-RACE approach using GeneRacer Kit (LifeTechnologies) according to the manufacturer's instructions. Finally the full coding regions were confirmed by RT-PCR using sequence specific primers obtained from the 5′- and 3′-RACE fragments, and proofreading *Pfu*-DNA polymerase (Promega). All PCR products were verified by sequencing after cloning into the pCR-BluntII vector (LifeTechnologies), and sequence data were submitted to GenBank (accession no. JX896426, *BjSultr1;1*; JX896427, *BjSultr1;2a*; JX896428, *BjSultr1;2b*; JX896429, *BjSultr1;2c*).

Sequence analyses were performed using ClustalW and neighbor-joining trees were generated using MEGA 5.05 [28].

### Gene expression analysis

Semi-quantitative RT-PCR analyses of *BjSultr1;1* and *BjSultr1;2 pool* were performed on first-strand cDNA deriving from total RNA extracted from roots. PCR was carried out for 24 cycles, where cDNAs were exponentially amplified by *Pfu-*DNA polymerase (Promega), using the following couples of primers: BjSultr1;1_dir_ 5'-ACGGAGGAGGGTCCGATGCAA-3' and BjSultr1;1_rev_ 5'-TTCGGGTCGATCACGGCCTGGCA-3' (producing a 453 bp fragment), BjSultr1;2_dir_ 5'-GGTTTTCGATTGGGGACGTA-3' and BjSultr1;2_rev_ 5'-TGTCAAGAGAACAACGATTGAC-3' (producing 1046 bp overlapping fragments). cDNA loading was normalized using the *BjTub* 846 bp amplicon (accession no. JX896430), as an internal control, obtained with primers designed on conserved regions of beta tubulin *Tub9* sequences of *Arabidopsis thaliana* (TAIR accession no. At4g20890) and *Brassica napus* (GenBank accession no. AF258790) as follow: Tub_dir_ 5′-TGTTGTGAGGAAGGAAGCTGAG-3′ and Tub_rev_ 5′-TCCTGTGTACCAATGAAGG-3′. PCR products were separated in agarose gels and stained with SYBR Green I (LifeTechnologies); signals were detected using a laser scanner (Typhoon 9200, GE Healthcare) with a 532 nm laser and a 526 nm filter.

For semi-quantitative RT-PCR analyses of the three different variants of *BjSultr1;2*, the entire ORFs were amplified with BjSultr1;2_ATG_ 5′-ATGTCTGGGAGAGCTCATCCTG-3′ and BjSultr1;2_STOP_ 5′-TCAGACCTCGTCGGAGAGTTTTG-3′ primers (producing a fragment of 1968 bp for *BjSultr1;2a* and fragments of 1959 bp for *BjSultr1;2b* and *BjSultr1;2c*). PCR products were then digested with ClaI endonuclease at 37°C for 3 h, and restriction products were separated in agarose gels. Signals were detected after staining as above described, and densitometrically analyzed using ImageJ 1.46 software [29].

All the expression analyses were performed using three independent cDNAs deriving from three independent experiments in which six plants were pooled for RNA extraction. Each cDNA was amplified, digested, run on gel, and quantified three times (*n* = 9).

### Heterologous expression of sulfate transporters and kinetic analysis in yeast

EcoRI-ended fragments, resulting from the amplification of *BjSultr1;1*, *BjSultr1;2a/b/c*, *ZmST1;1*, and *AtSultr2;1* ORFs using appropriate primers (BjSultr1;1_KATG_ 5′-CACTAGAATTCTAAAAAATGGCCAAGACTAATCCGCCGGA-3′ and BjSultr1;1_KSTOP_ 5′-TGACCGAATTCTTATGCTTGTTGCTCAGCCAAT-3′, BjSultr1;2_KATG_ 5′-CACTAGAATTCTAAAAAATGTCTGGGAGAGCTCATCCTG-3′ and BjSultr1;2_KSTOP_ 5′- TGACCGAATTCTCAGACCTCGTCGGAGAGTTTTG-3′, ZmST1;1_KATG_ 5′-CAGCGAATTCTAAAAAATGCCGCCGCGAACGGTGTCC-3′ and ZmST1;1_KSTOP_ 5′-GCGCGAATTCTCAGACATTATCGACCATCTTAGGAGC-3′, and AtSultr2;1_KATG_ 5′-CAGCGAATTCTAAAAAATGAAAGAGAGAGATTCAGAGA-3′ and AtSultr2;1_KSTOP_ 5′-TGACCGAATTCTTAAACTTTTAATCCAAAGCAAGCATCAA-3′) including a consensus sequence for translation initiation in yeast [30], were subcloned in the EcoRI site of the yeast (*Saccharomyces cerevisiae*) expression vector pESC-TRP (Stratagene) under the control of *GAL10* promoter. Chimeric and empty vectors were used to transform the yeast double sulfate transporter mutant CP154-7A (*MATα his3 leu2 ura3 ade2 trp1 sul1*:*LEU2 sul2*:*URA3*) [31] using the standard lithium acetate method [32], and Trp^+^ recombinant yeast cells were selected. Complementation tests were performed as previously described [9].

For the growth analysis, recombinant yeast cells were grown – at 28°C in a synthetic Trp-free liquid medium containing yeast nitrogen base and required amino acids – up to reach a mid-log phase. Yeast cells were then washed twice with sterile distilled water and resuspended to a final absorbance of 0.1 *A*_600_ unit in the B minimal medium [33], supplemented with 40 μg mL^-1^ adenine and 200 μg mL^-1^ histidine to meet the auxotrophies of the strain, and containing different amounts of Na_2_SO_4_ or 100 μM DL-homocysteine (HCys) as sole sulfur sources. Yeasts were incubated at 28°C and growth was monitored by measuring the absorbance at 600 nm. At the end of the growing period, 30 mL of the yeast culture was harvested, washed twice in sterile distilled water, resuspended in 4 mL of boiling buffered ethanol (75% ethanol in 10 mM HEPES, pH 7.1) and incubated for 3 min at 80°C. After cooling down the mixture on ice, the volume was reduced by evaporation at 70°C, the residue was resuspended in 4 mL of distilled water and centrifuged for 15 min at 13000 *g* and 4°C. The supernatant was collected and the sulfate content was then determined according to the turbidimetric method described by Tabatabai and Bremner [34].

The duplication times of the yeast cells were calculated by fitting the equation *A*_600_(*t*) = *A*_600_(*t*_0_) *e*^kt^ to the experimental data. The growth constant (k_G_) was estimated by expressing the growth rates (dt^-1^) of complemented yeasts as a function of sulfate concentrations in the media, and by fitting the Michaelis-Menten equation to the data.

### Determination of thiols, sulfate and cadmium content

Roots and shoots were pulverized using mortar and pestle in liquid N_2_. Total nonprotein thiols (NPTs) and Cd contents were determined according to Nocito and co-workers [35]. Total GSH was measured according to Griffith [36].

Sulfate was extracted by homogenizing the samples in 1:10 (w/v) ice-cold 0.1 N HNO_3_. After heating at 80°C for 40 min, the extracts were filtered and the sulfate contents were determined according to the turbidimetric method described by Tabatabai and Bremner [34].

### Sulfate influx assay and analysis of root-to-shoot sulfate translocation

Sulfate influxes into the roots were measured by determining the rates of ^35^S uptake, over a 15 min pulse in incubation solutions labeled with the radiotracer. Briefly, a single plant was placed onto 400 mL of a fresh acclimation solution, containing 200 μM MgSO_4_, supplemented or not with CdCl_2_ at different concentrations, aerated and thermoregulated at 26°C. Radioactive pulses were started by adding ^35^S-labeled Na_2_SO_4_ to the uptake solutions. Specific activity was 4.7 kBq μmol^-1^. At the end of the pulse period, roots were excised from shoots, rinsed twice for 1 min in 400 mL of a 4 mM CaSO_4_ nonradioactive solution at 4°C, blotted with paper towels, weighed, and then heated for 20 min at 80°C in 0.1 N HNO_3_ (10 mL g^-1^ fresh weight). Radioactivity was measured on aliquots of the extracting solution by liquid scintillation counting in a β counter (LS 6000SC, Beckman).

For the analysis of root-to-shoot sulfate translocation, shoots were cut at 2 cm above the roots with a microtome blade. Xylem sap exuded from the lower cut surface was collected by trapping into a 1.5 mL plastic vial filled with a small piece of cotton for 1.5 h. The amount of collected sap was determined by weighing and the sulfate concentration was then determined according to the turbidimetric method described by Tabatabai and Bremner [34].

### Statistical analysis

Statistical analysis was carried out using SigmaPlot for Windows version 11.0 (Systat Software, Inc.). Quantitative values are presented as mean ± standard error of the mean (SE). Significance values were adjusted for multiple comparisons using the Bonferroni correction. Statistical significance was at *P* < 0.05. Student’s *t*-test was used to assess the significance of the observed differences between control and treated plants. Statistical significance was at *P* ≤ 0.001.

## Results

### Cloning and functional characterization of four high-affinity sulfate transporter cDNAs

Plant sulfate transporters are encoded by a multi-gene family whose members have specific functions in sulfate acquisition, systemic distribution and subcellular localization [37-39]. In this work we identified four sulfate transporter cDNAs expressed in *B. juncea* roots: one named *BjSultr1;1*, and three, with closely related sequences, named *BjSultr1;2a*, *BjSultr1;2b* and *BjSultr1;2c*. All the cDNA-encoded proteins were predicted as putative high-affinity sulfate transporters belonging to the group 1 of the sulfate transporter family (Additional file 1). Sequence analyses revealed that the amino acid identities of these proteins with those of Arabidopsis belonging to the same cluster were 86% (BjSultr1;1 *vs* AtSultr1;1) and 94% (BjSultr1;2a/b/c *vs* AtSultr1;2), suggesting that the *B. juncea* and Arabidopsis sulfate transporters would share functions in mediating root sulfate uptake. In such a way *BjSultr1;1* could be considered the ortholog of *AtSultr1;1*, whereas the three *BjSultr1;2* cDNAs would represent three orthologous variants of *AtSultr1;2*.

Concerning the three *BjSultr1;2* forms, some additional data need to be taken into account. Sequence analysis (Additional file 2; Additional file 3) revealed that the coding sequence of the longer variant, *BjSultr1;2a*, shares 98% of nucleotide identity with *Bra015641*, a gene encoding a Sultr1;2 form on the chromosome A7 of *B. rapa* – one of the two parents of *B. juncea* of which the genome has been recently sequenced [23] – and only 91% of nucleotide identity with *Bra008340*, a second form of Sultr1;2 found on the chromosome A2 of *B. rapa*. On the other hand the coding sequences of the shorter variants, *BjSultr1;2b* and *BjSultr1;2c*, share the highest identities with *Bra008340* (95% and 99%, respectively). Unfortunately, we failed in finding any information about the sulfate transporter genes of *B. nigra* (the other *B. juncea* parent) in public genomic databases.

The heterologous expressions of *BjSultr1;1*, *BjSultr1;2a*, *BjSultr1;2b*, and *BjSultr1;2c* in the yeast (*Saccharomyces cerevisiae*) double sulfate transporter mutant CP154-7A [31] were able to revert the yeast mutant phenotype, allowing it to grow on a minimal medium containing 100 μM Na_2_SO_4_ as a sole sulfur source (Additional file 4), confirming the identity of these *B. juncea* clones as functional sulfate transporters.

**Figure 1:**
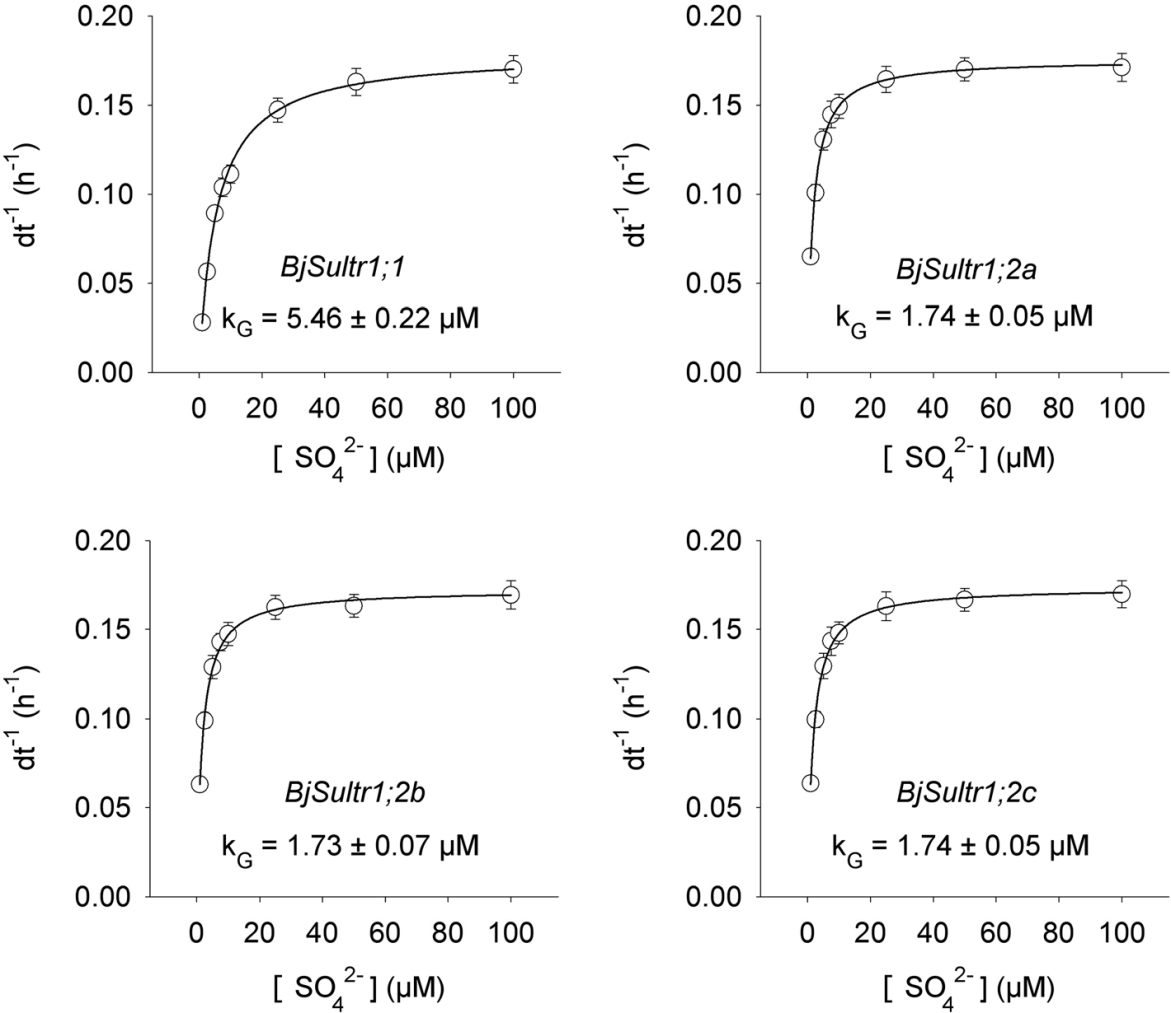
**Estimation of the growth constant (k**_**G**_**) dependent on sulfate of the yeasts expressing the *Brassica juncea *sulfate transporters.** The duplication times (dt) of the complemented yeast cells were calculated by fitting the equation *A*_600_(*t*) = *A*_600_(*t*_0_) *e*^kt^ to the experimental data reported in Additional file 5. k_G_ was estimated by expressing the growth rates (dt^-1^) of complemented yeasts as a function of sulfate concentrations in the media, and by fitting the Michaelis-Menten equation to the data. Data points and error bars are means and SE of two experiments run in triplicate (*n* = 6).

In order to estimate the apparent k_M_ for sulfate of each transporter we first analyzed the growth curves of complemented yeasts incubated in liquid media containing different sulfate concentrations (from 0 to 100 μM) as sole sulfur sources (Additional file 5). In these conditions the amount of sulfate taken up by the transporter and available for metabolic assimilation should be expected to be the main limiting factor for yeast growth. If this were not the case – i.e. if some enzymatic activities along the pathways of sulfate assimilation or Cys consumption would limit yeast growth – a gradual accumulation of non-assimilated sulfate into the yeast cells should be expected. As detailed in Additional file 6, the sulfate content of complemented yeast cells, measured in the mid-log phase, did not change in the range of 1–100 μM sulfate external concentration, and the growth rate of the cells incubated in minimal media containing an organic sulfur source (100 μM DL-homocysteine; HCys) was higher than those measured at the highest sulfate external concentration analyzed. Moreover, sulfate concentration in the yeast cells incubated in the absence of sulfate was always lower than 0.05 nmol A_600_ ^-1^. From these data we can reasonably conclude that, at least in our conditions, the yeast growth rate is limited by sulfate uptake and fits the rate of sulfate influx through the single heterologously expressed sulfate transporter. Thus, by expressing the growth rate values as a function of sulfate concentrations we can calculate a growth constant, k_G_, defined as the sulfate concentration at which half of the maximum yeast growth rate is reached. As shown in Additional file 7, such a constant allows us to discriminate high- and low-affinity sulfate transporters, since it is closely related to the apparent k_M_ for sulfate of the transporters. Least square fittings (Figure 1) revealed that the growing isotherms of the four complemented yeasts can be properly described by single hyperbolic Michaelis-Menten functions, with k_G_ for sulfate in the micromolar range, indicating that these proteins are high-affinity sulfate transporters; in particular the k_G_ values were 5.46 ± 0.22 μM (BjSultr1;1), 1.74 ± 0.05 μM (BjSultr1;2a), 1.73 ± 0.07 μM (BjSultr1;2b), and 1.74 ± 0.05 μM (BjSultr1;2c).

### Effect of Cd exposure and sulfate limitation on sulfate uptake and sulfur allocation in *Brassica juncea* plants

All the data presented in this paragraph derived from experiments aimed at comparing environmental conditions (Cd exposure and sulfate limitation) in which sulfate uptake induction should occur. For these purposes plants were exposed to 10 and 25 μM Cd^2+^ for 2 days or grown under sulfate limitation (50 and 10 μM SO_4_ ^2-^) for a 10-day period. Control plants were grown at 200 μM SO_4_ ^2-^ in the absence of Cd.

Cadmium exposure neither significantly influenced the growth of shoots and roots, nor produced any apparent symptom of stress; conversely, lowering sulfate concentration in the growing solution significantly increased root growth without affecting shoots, as indicated by the values of the shoot/root ratio which decreased from 3.82 to 2.20 (Table 1). The total amount of Cd retained by roots increased as the metal concentration in the external medium did, whereas it reached similar values in the shoots of plants grown at 10 and 25 μM Cd^2+^ (Table 1).

**Table 1:** Dry weight of roots and shoots and Cd accumulation

**Experimental condition**	**Dry weight**		**Shoots/Roots**	**Cd**^**2+ **^**content**	
	**Roots**	**Shoots**		**Roots**	**Shoots**
	**g**			**μmol g**^**-1 **^**DW**	
Control	0.063 ± 0.003 (a)	0.241 ± 0.010 (a)	3.82	ND	ND
10 μM Cd^2+^	0.069 ± 0.003 (a)	0.252 ± 0.011 (a)	3.65	25.81 ± 1.18 (a)	5.66 ± 0.25 (a)
25 μM Cd^2+^	0.066 ± 0.004 (a)	0.231 ± 0.012 (a)	3.50	97.33 ± 3.99 (b)	5.00 ± 0.22 (a)
50 μM SO_4_^2-^	0.112 ± 0.005 (b)	0.251 ± 0.011 (a)	2.24	ND	ND
10 μM SO_4_^2-^	0.110 ± 0.005 (b)	0.243 ± 0.011 (a)	2.20	ND	ND

Plants were exposed to different Cd concentrations (10 and 25 μM) for 48 h or grown under different sulfate concentrations (50 and 10 μM) for 10 days. Control plants were grown under 200 μM SO_4_ ^2-^ and were not exposed to Cd. Cadmium content was measured by ICP-MS. Values are means ± SE of three experiments run in triplicate (*n* = 9). Different letters indicate significant differences (*P* < 0.05). ND, not detectable.

**Figure 2:**
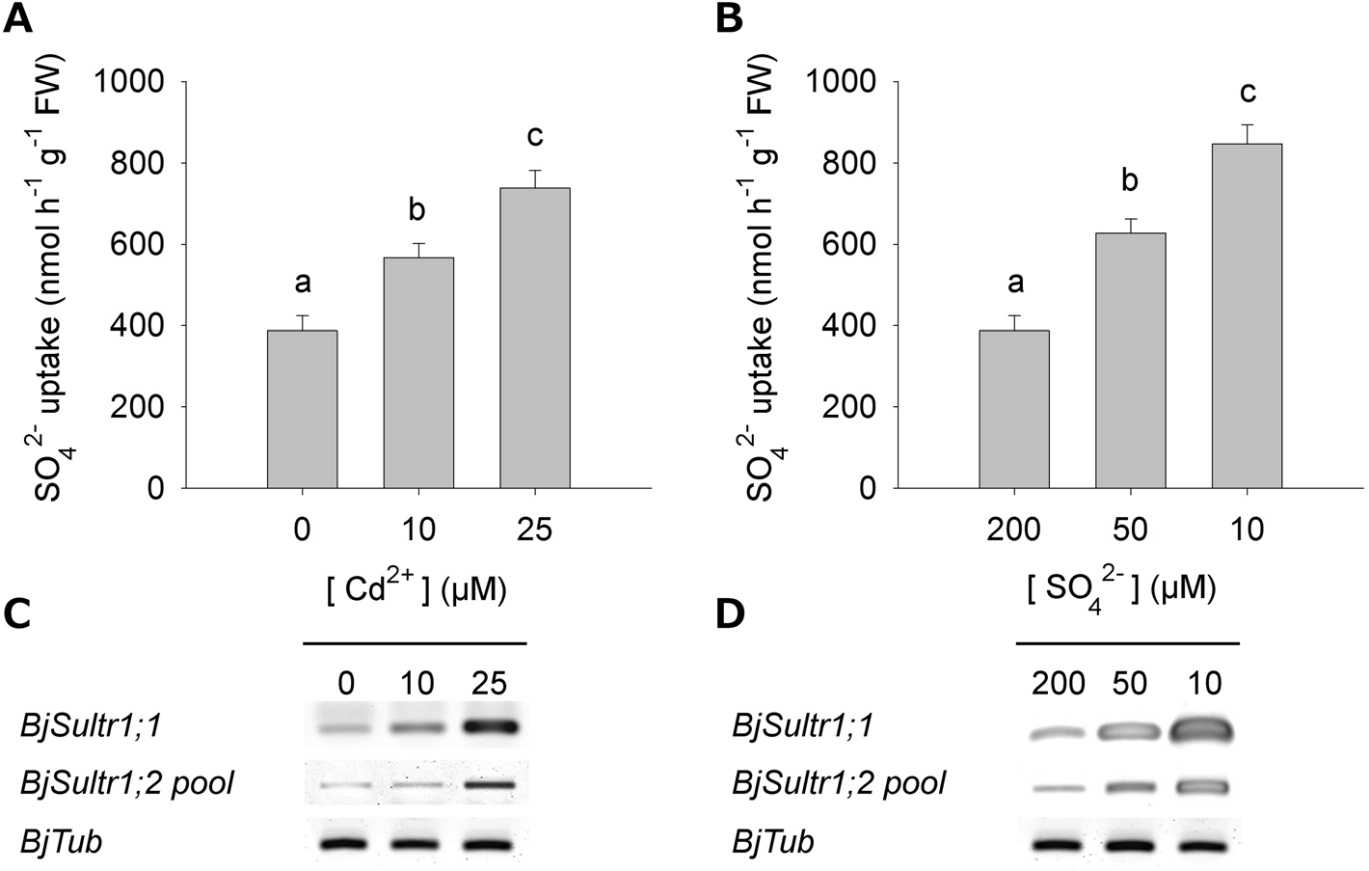
**Changes in sulfate uptake capacity of *Brassica juncea *roots.** Plants were exposed to different Cd concentrations for 48 h **(A, C)** or grown under different sulfate concentrations for 10 days **(B, D)**. **(A, B)** Sulfate influxes were evaluated by measuring the rate of ^35^SO_4_ ^2-^ absorption into roots of intact plants over a 15 min pulse. The incubation solutions contained 200 μM SO_4_ ^2-^. Bars and error bars are means and SE of three experiments run in triplicate (*n* = 9). Different letters indicate significant differences (*P* < 0.05). **(C, D)** Semi-quantitative RT-PCR analysis of *BjSultr1;1* and *BjSultr1;2* gene expression. PCRs were carried out for 24 cycles where cDNAs were exponentially amplified. For *BjSultr1;2 pool*, primers were designed on conserved sequences of the three *BjSultr1;2* variants, and gave overlapping amplification products of 1046 bp. PCR products were separated in agarose gel and stained with SYBR Green I. Signals were detected using a laser scanner with 532 nm laser and 526 nm filter. *BjTub*, tubulin. A representative set of data from three independent experiments is given.

Cadmium exposure and sulfate limitation deeply affected the sulfate uptake capacity of the root, as indicated by the values of ^35^S-sulfate uptake, measured at 200 μM SO_4_ ^2-^ external concentration (Figure 2A, B). In Cd exposed plants the rate of sulfate uptake increased up to 0.9-fold with respect to the untreated control at the highest Cd external concentration (25 μM). Similar behaviors were observed in sulfur-starved plants, in which the rate of sulfate uptake increased as the sulfate concentration in the external medium decreased, reaching values 1.2-fold higher than in sulfur-sufficient control (200 μM SO_4_ ^2-^). These trends were closely associated to changes in the transcript level of *BjSultr1;1* and in the cumulative amount of the three transcripts of the *BjSultr1;2* variants (*BjSultr1;2 pool*), which significantly accumulated as the severity of the stresses increased (Figure 2C, D).

Taken as a whole these preliminary results indicate that 48 h Cd exposure and 10-day sulfate limitation produced similar induction of sulfate uptake. Since such effects should presumably be related to changes in the sulfur nutritional status of the plants, we analyzed the levels of NPTs, GSH and sulfate of both roots and shoots, assuming the pools of these intermediates as the main diagnostic indicators of the sulfur nutritional status.

**Figure 3:**
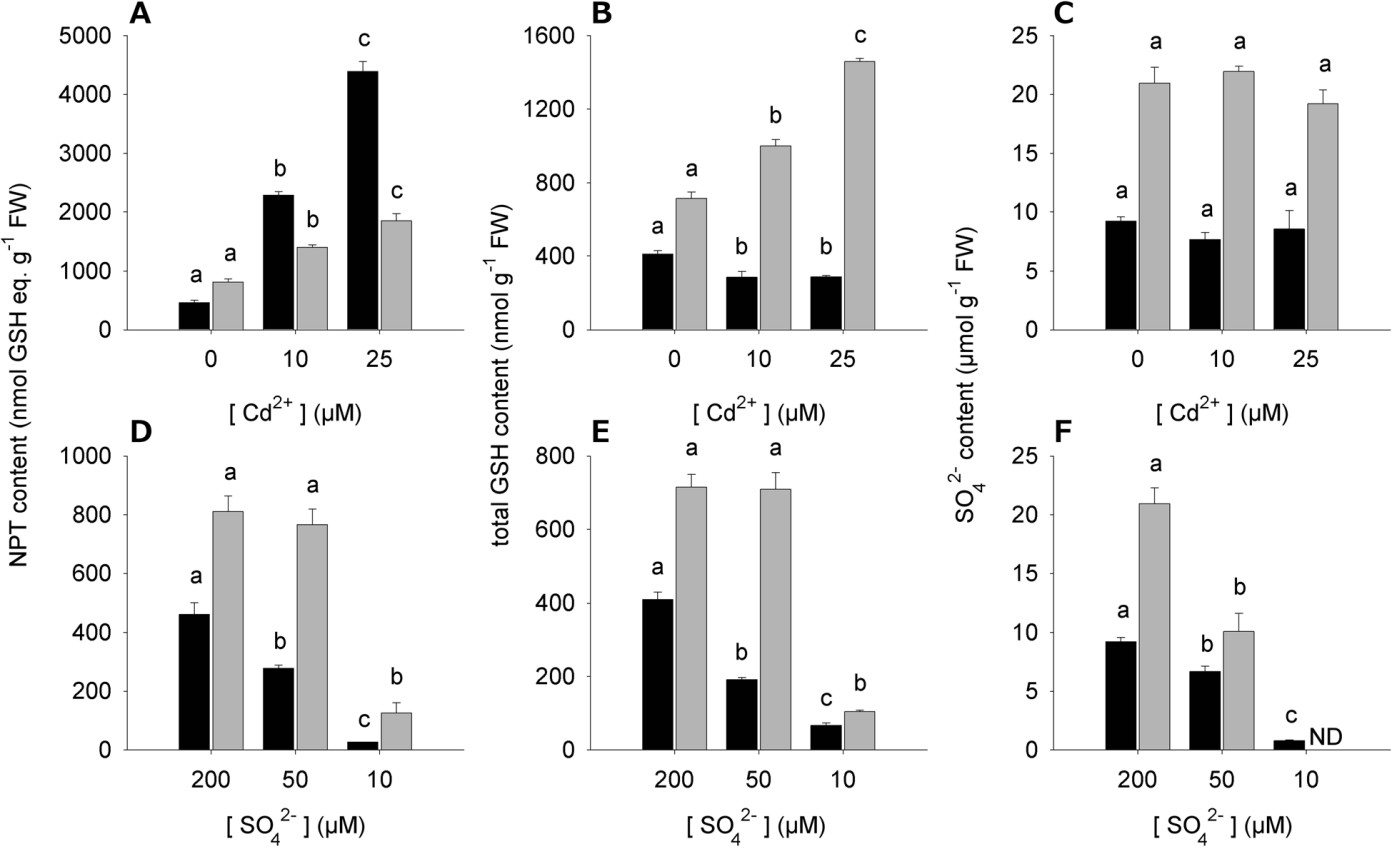
**Effects of Cd exposure and sulfate limitation on the sulfur nutritional status of *Brassica juncea *plants.** Plants were exposed to different Cd concentrations for 48 h **(A, B, C)** or grown under different sulfate concentrations for 10 days **(D, E, F)**. **(A, D)** NPT contents of roots (black bars) and shoots (grey bars) are expressed as GSH equivalents. **(B, E)** Total GSH contents of roots (black bars) and shoots (grey bars). **(C, F)** Sulfate contents of roots (black bars) and shoots (grey bars). Bars and error bars are means and SE of three experiments run in triplicate (*n* = 9). Different letters indicate significant differences between treatments (*P* < 0.05). ND, not detectable.

Cd exposure produced significant changes in the NPT levels of the root, which progressively increased as Cd concentration in the external medium did (Figure 3A), whilst at the same time a decrease of the total GSH pools was observed (about 30% with respect to the control in all analyzed conditions; Figure 3B). Such a trend was probably related to PC biosynthesis and accumulation according to the progressive increase in Cd root content (Table 1). The sulfate pools of the root were not affected by Cd exposure (Figure 3C). Quite similar behaviors were observed in the shoots of Cd exposed plants, since the NPT levels increased with Cd concentration in the external medium and the sulfate concentration was not affected by the presence of the metal; however, a Cd-dependent increase in the GSH levels was observed (Figure 3A, B, C).

**Figure 4:**
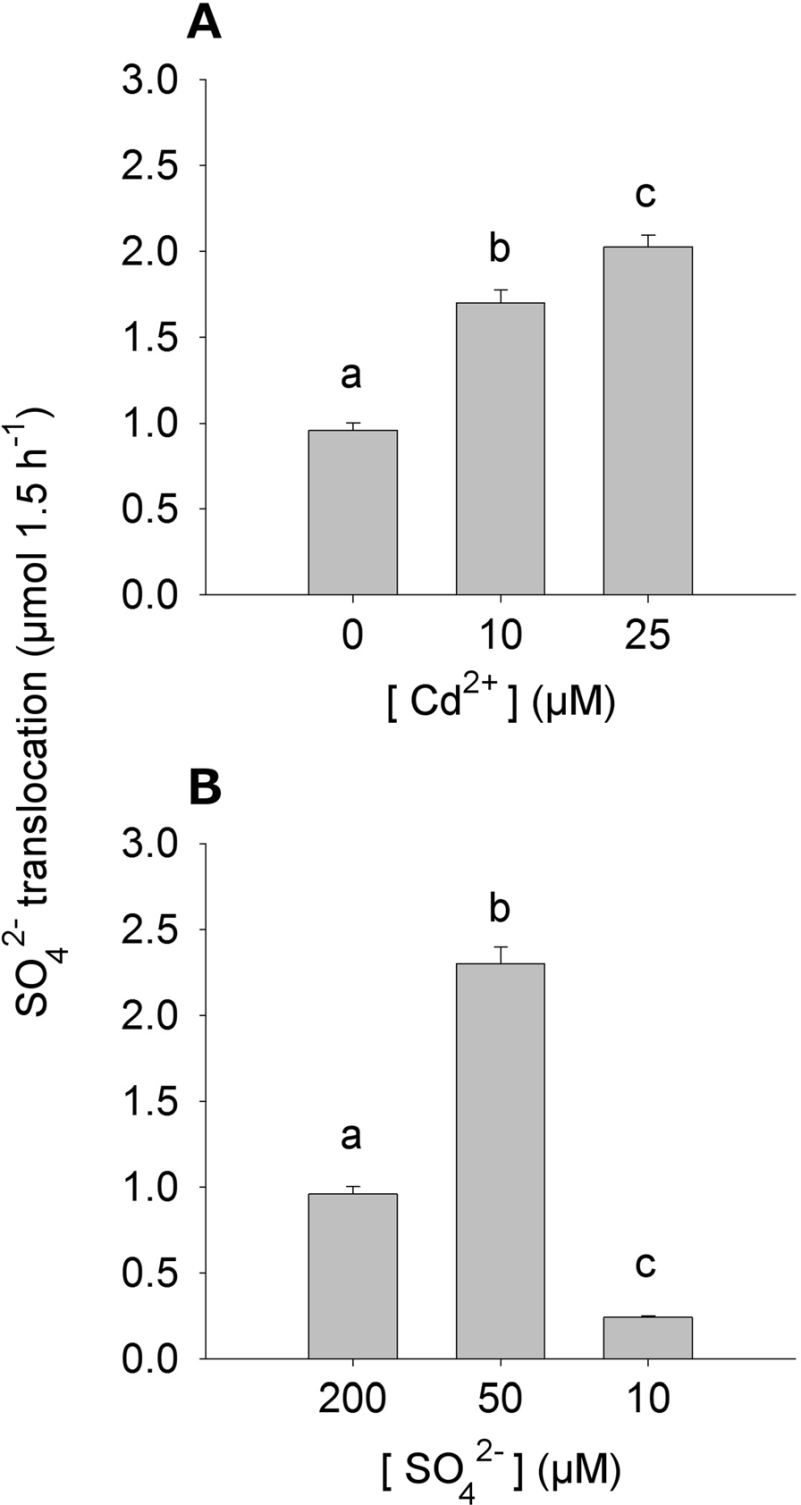
**Effects of Cd exposure and sulfate limitation on sulfate translocation in *Brassica juncea *plants.** Plants were exposed to different Cd concentrations for 48 h **(A)** or grown under different sulfate concentrations for 10 days **(B)**. At the end of the experimental periods, shoots were separated from roots and the xylem sap exuded from the cut (root side) surface was collected to be analyzed for sulfate content. Bars and error bars are means and SE of three experiments run in triplicate (*n* = 9). Different letters indicate significant differences (*P* < 0.05).

As expected, a stepwise contraction in the levels of all the diagnostic indicators was observed in the root of plants grown for 10 days under sulfate limitation. Indeed, NPT, GSH and sulfate contents measured in the root tissues dramatically decreased as sulfate availability in the external medium did (Figure 3D, E, F). Following sulfate limitation, sulfate content of the shoot steadily decreased, reaching the minimal value at 10 μM SO_4_ ^2-^ external concentration; differently, NPT and GSH levels did not significantly change when we lowered sulfate external concentration from 200 to 50 μM, whilst a sharp decrease in the level of these compounds was observed by moving toward the lowest (10 μM) sulfate concentration analyzed (Figure 3D, E, F).We also analyzed the dynamic of root-to-shoot sulfate translocation by measuring the concentration of the anion in the xylem sap of Cd-exposed or sulfur-starved plants. In these experiments, sulfate translocation was estimated as the amount of sulfate ions loaded and transported in the xylem sap for 1.5 h. Results indicate that the amount of sulfate ions transported in the xylem sap progressively increased following Cd exposure (Figure 4A); differently, sulfate translocation increased when shifting sulfate external concentration from 200 to 50 μM, and sharply decreased when moving toward the lowest (10 μM) sulfate concentration analyzed (Figure 4B).

### Quantitative analysis of the expression of the three *BjSultr1;2* variants

Since the three *BjSultr1;2* forms are not polymorphic enough to be distinguished by means of a simple PCR (Additional file 8), we developed a suitable method to study changes in their expression by coupling semi-quantitative RT-PCR analysis with the use of an opportune restriction enzyme.

**Figure 5:**
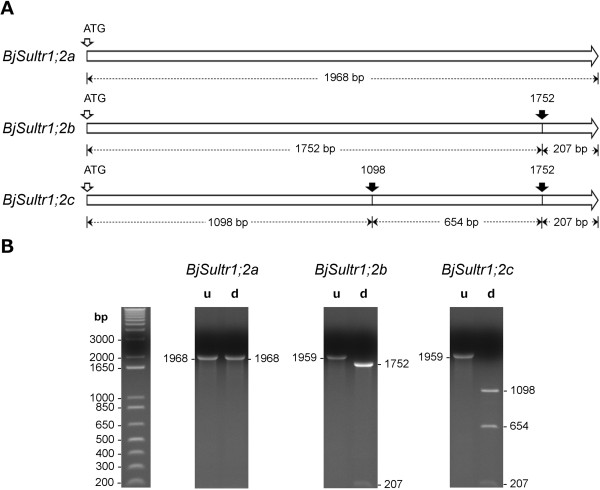
**Restriction analysis of the three *BjSultr1;2 *cDNAs. (A)** The three *BjSultr1;2* variants have restriction site polymorphisms for the Cla I endonuclease. Black arrows indicate the relative position of Cla I restriction sites in each open reading frame. The expected lengths of the restriction fragments obtained after digestion with ClaI are indicated. **(B)** Characteristic restriction patterns obtained from the digestion of each cDNA with ClaI. Single cDNAs were obtained by PCR using a recombinant plasmid, containing a unique *BjSultr1;2* clone, as template. u, undigested; d, digested.

Sequence analysis revealed that the three *BjSultr1;2* cDNAs have restriction site polymorphisms for the ClaI endonuclease, which enabled us to discriminate the three variants after digestion. As detailed in Figure 5A: i) *BjSultr1;2a* (1968 bp) is not cut by ClaI; ii) *BjSultr1;2b* (1959 bp) is cut by ClaI 1752 bp downstream of the start codon; iii) *BjSultr1;2c* (1959 bp) is cut twice by ClaI, 1098 and 1752 bp downstream of the start codon. As a consequence the digestion of the cDNA clones with ClaI produces characteristic restriction patterns with some diagnostic bands useful to discriminate the three forms (Figure 5B). The characteristic undigested 1968 bp band is a diagnostic marker of *BjSultr1;2a* presence, the 1752 bp fragment only results from the digestion of *BjSultr1;2b*, whilst both the 1098 and 654 bp bands are specifically produced following the digestion of *BjSultr1;2c*. Finally the 207 bp band is a digestion product shared among *BjSultr1;2b* and *BjSultr1;2c*, and therefore does not give any result useful for our purposes.

Starting from this rationale, we designed a couple of primers amplifying at the same time the entire open reading frames of the three clones with the same efficiency (data not shown), and we used these oligos for the semi-quantitative RT-PCR analysis of the effects of 48 h exposure to 25 μM Cd^2+^ or 10-day sulfate limitation (10 μM SO_4_ ^2-^) on the expression of the three *BjSultr1;2* variants. A restriction analysis using the ClaI endonuclease followed the amplification reactions.

**Figure 6:**
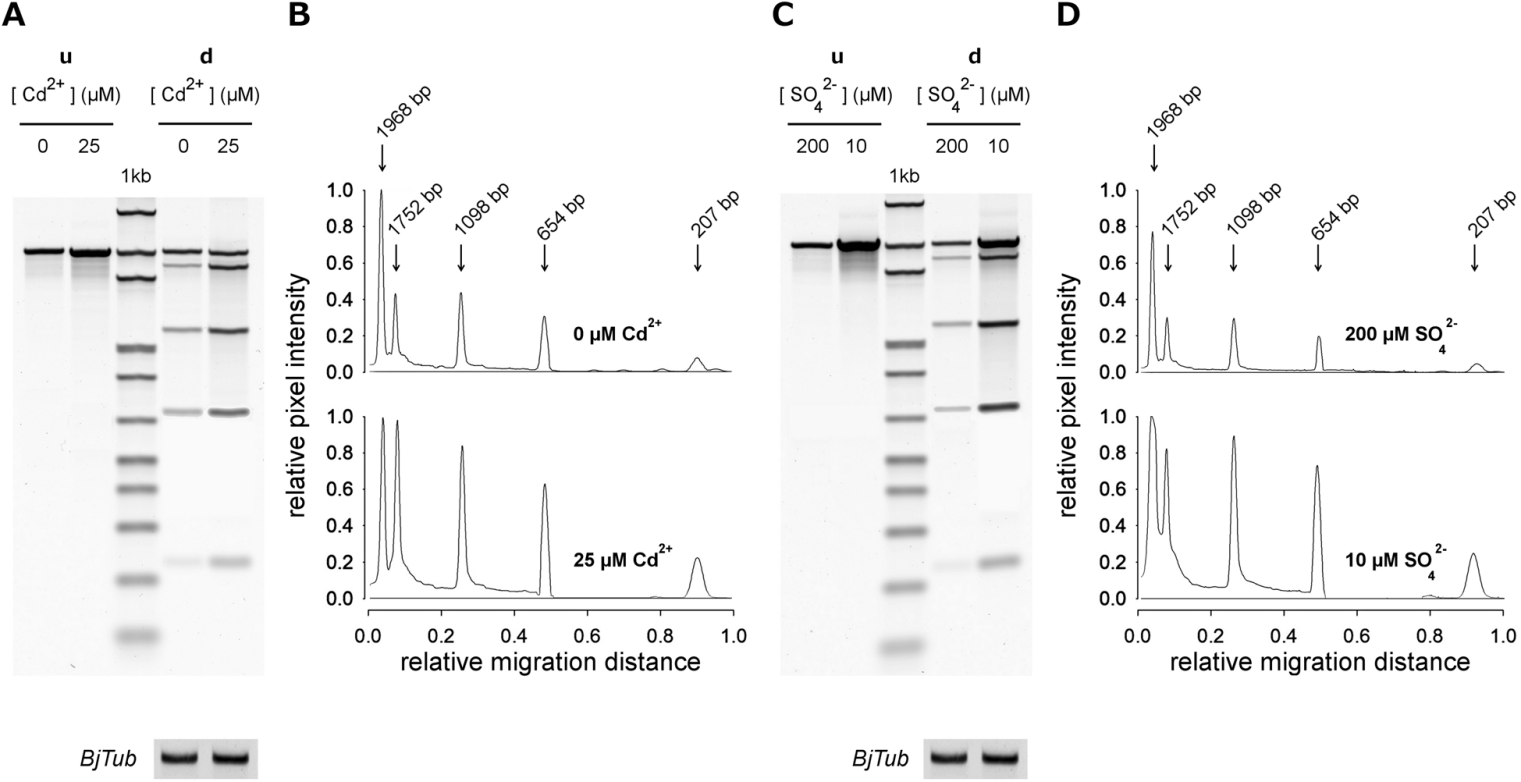
**RT-PCR analyses of the three *BjSultr1;2 *forms in the roots of *Brassica juncea *grown under Cd exposure or sulfate limitation.** Plants were exposed to 25 μM Cd^2+^ for 48 h **(A, B)** or grown under 10 μM SO_4_ ^2-^ for 10 days **(C, D)**. **(A, C)** The entire ORFs of the three *BjSultr1;2* forms were amplified and PCR products, digested (d) or not digested (u) with ClaI endonuclease, were electrophoresed on agarose gel and stained with SYBR Green I. cDNA loading was normalized using *BjTub* as an internal control. Signals were detected using a laser scanner with 532 nm laser and 526 nm filter. **(B, D)** Densitometric analysis. Arrows indicate the relative position of each electrophoretic band obtained after digestion of PCR products with ClaI. A representative set of data from three independent experiments is given. For statistical analysis, see Additional file 9.

Results show that the cumulative amount of the *BjSultr1;2* transcripts in the roots was significantly higher in Cd exposed plants than in the untreated control ones (+217%, Figure 6A) as already shown in Figure 2C. Such a behavior resulted from changes in the expression of *BjSultr1;2b* and *BjSultr1;2c* only (Figure 6A). In fact, the densitometric analysis of each diagnostic band indicated that *BjSultr1;2b* and *BjSultr1;2c* transcript levels significantly increased by 585% and 301%, respectively, whilst the *BjSultr1;2a* expression was not affected by Cd exposure (Figure 6B, Additional file 9). Similar behaviors were observed by analyzing changes in the expression pattern of the three *BjSultr1;2* forms in plants exposed for 48 h to a lower (10 μM) Cd concentration (Additional file 10). In fact, also in this condition the response to Cd, though to a lesser extent, was only ascribable to specific increases in the relative amount of *BjSultr1;2b* (+150%) and *BjSultr1;2c* (+75%) transcript.

By contrast, in the case of sulfate limitation, the increase in the cumulative amount of the *BjSultr1;2* transcripts (+455% with respect to the sulfur-sufficient control) was ascribable to changes in the transcript levels of all the three forms (Figure 6C). In particular, the *BjSultr1,2a*, *BjSultr1;2b*, and *BjSultr1;2c* transcript levels significantly increased by 371%, 483%, and 618%, respectively (Figure 6D, Additional file 9).

## Discussion

*Brassica juncea* (L.) Czern & Coss (AABB, *n* = 18) is believed to have originated from the interspecific hybridization of two base “diploid” genomes provided by *Brassica rapa* L. (AA; *n* = 10) and *Brassica nigra* L. (BB; *n* = 8) [24,40]. Both the diploid parents are thought to be ancient polyploids since they still exhibit highly replicated genomes, each containing three paralogous subgenomes closely related to that of *Arabidopsis thaliana *[21-23]. In spite of the whole-genome triplication event – thought to have occurred between 13 and 17 million years ago – most comparative studies have shown that the number of each ancestral gene retained in the genome of the modern diploid *Brassica* is variable, since paralogous regions exhibit interspersed gene losses and insertions. Interestingly, in the recently sequenced *B. rapa* genome the extent of gene loss among triplicated genome segments varies, with one of the three copies consistently retaining a disproportionately large fraction of the genes expected to have been present in its ancestor [23]. Such evolutionary events are thought to be the biological basis of the immense plasticity of *Brassica* species and may have led to a diversification of the genes retained in more than one copy, in terms of function and/or expression. Searching for orthologs of the Arabidopsis high-affinity sulfate transporter genes involved in sulfate uptake and retained in the genome of *B. rapa* – one of the two parents of *B. juncea* – revealed the existence of three distinct loci, annotated as *Bra022623*, *Bra015641* and *Bra008340*. The first locus encodes for a putative high-affinity sulfate transporter closely related to Arabidopsis *AtSultr1;1*, whilst the other two loci encode for two different forms of a high-affinity sulfate transporter functional related to Arabidopsis *AtSultr1;2*, indicating these gene loci as paralogs. As expected, a much more complex picture was found in the allopolyploid *B. juncea* in which we were able to identify an orthologous form of *AtSultr1;1* (*BjSultr1;1*) and three orthologous forms of *AtSultr1;2* (*BjSultr1;2a/b/c*). From the results obtained by the sequence analysis, and in the absence of any other information so far available about the *B. nigra* genome, we can reasonably suppose: i) *BjSultr1;2a* as the ortholog of *Bra015641* on the genome A or B of *B. juncea*; ii) *BjSultr1;2b*/*c* as allelic variants orthologous of *Bra008340* on the A or B genome of *B. juncea* or a homeologous gene pair related to *Bra008340* on the A and B genomes of *B. juncea*. Moreover, since the progeny derived from self-fertilization inherited all the three *BjSultr1;2* variants (data not shown), it seems likely to exclude that *BjSultr1;2b* and *BjSultr1;2c* would be allelic, making plausible the hypothesis they are instead present at different homeologous gene loci on A and B genomes, each in homozygous configuration; otherwise, a simple mendelian segregation would be observed. In any case, since the three *BjSultr1;2* forms would share a common ancestor gene, they may either have retained their original functions and expressions, or – as it is often the case – have accumulated deleterious mutations or have evolved novel gene interactions through the processes of sub-functionalization and/or neo-functionalization [25,26].

Results of complementation tests in the yeast mutant strain CP154-7A, defective in its two sulfate transporters and thus unable to grow on media containing low concentrations of sulfate as the sole sulfur source [31], proved the capacity of BjSultr1;1 and BjSultr1;2a/b/c to transport sulfate ions across the plasma-membrane (Additional file 4). Moreover, kinetic analysis of the growth (G) isotherms of complemented yeasts, revealed that BjSultr1;1 and BjSultr1;2a/b/c have high affinities for sulfate, as revealed by the k_G_ values similar to the apparent k_M_ values of other plant high-affinity sulfate transporters [9,41-44] indicating that all the *B. juncea* clones have retained their functions. It is also worthy of note that the sulfate transporter BjSultr1;1 has an apparent affinity for sulfate (k_G_ = 5.46 μM) three times lower than those of the three BjSultr1;2 forms (Figure 1). Finally, phylogenetic analyses indicate these transporters as functionally related to AtSultr1;1 and AtSultr1;2 and then we can infer their probable function in mediating root sulfate uptake from the soil solution [14,43,44].

Physiological analysis reveals that Cd exposure as well as sulfate limitation induces sulfate uptake in *B. juncea* roots. Such behaviors seem to be related to the induction of high-affinity sulfate transporters belonging to the group I, as indicated by the increase in the transcript levels of *BjSultr1;1* and *BjSultr1;2 pool* (Figure 2). Thus – at the physiological level – *B. juncea* also retains the typical responses of sulfate uptake to Cd or sulfur shortage [9,15]. The apparent quantitative discrepancy between the changes in the transcript levels of *BjSultr1;1* and *BjSultr1;2 pool* and the resulting increases in sulfate uptake may be due to additional regulatory mechanisms working in parallel with the transcriptional control of the high-affinity sulfate transporter genes [45].Considering the current model of demand-driven regulation of sulfate uptake, such inductions should be related to the sulfur nutritional status reached by plants in the two growing conditions. Changes in the amounts of sulfur-containing compounds that we assume as the main diagnostic indicators of the sulfur nutritional status of root and shoot clearly indicate that Cd exposure and sulfate limitation influence sulfur allocation throughout the whole plant, generating deeply different local nutritional states which make it difficult to individuate an unequivocal and common nutritional signal related to the expression of sulfate transporters (Figure 3). Indeed, as expected, lowering sulfate concentration in the external medium necessarily results in a significant contraction of all the analyzed sulfur pools of the roots, whilst Cd stress produces a typical increase in the level of NPTs, probably due to the activation of GSH-dependent PC biosynthesis, without affecting the sulfate content of the roots.

Root responses to Cd exposure seem to be due to homeostatic mechanisms driven by increases in the sulfur need of the plants, since, as previously reported [9], the effect of Cd on sulfate uptake capacity is closely related to the NTP levels of both root and shoot and then to the strength of the Cd-induced additional sink for thiols (Additional file 11). Under Cd exposure the NPT levels in plant tissues significantly increase, reaching values 8.5 (root) and 1.3 (shoot) fold higher than in the control at the highest concentration analyzed. Since the sulfate pools of both root and shoot seem not to be affected by Cd exposure, it appears clear that the additional sulfur required to sustain thiol biosynthesis necessarily derives from the activation of sulfate uptake. On the other hand, root responses to sulfate shortage appear likely to be dependent on the need for allocating the limiting nutrient in the best and most efficient way. In fact, the stepwise decrease in the sulfate content of the roots seems to be related not only to a decrease in sulfate availability, but also to a transient activation of sulfate translocation making shoots less sensitive to sulfate deficiency (Figure 4B) [46]. Noticeably, Cd exposure neither decreases root sulfate content (Figure 3C) nor inhibits root sulfate uptake (Figure 2A) and sulfate transporter gene expression (Figure 2C), but rather significantly enhances sulfate translocation (Figure 4A) and its metabolism as shown by the significant increases in the GSH levels of the shoot (Figure 3B). Such an effect should be related to the activation of both PC biosynthesis and mechanisms involved in controlling oxidative damage due to the accumulation of free Cd ions in the leaves [47,48]. Moreover, from these results we can also speculate that the over-accumulation of GSH in the shoot could help roots in detoxifying Cd through reallocating mechanisms involving phloem translocation, as previously reported in other species [49,50]. In this way, the excess of sulfate taken up by roots would partly bypass root assimilation to be directly metabolized in organs less affected by Cd stress, without however affecting the root sulfate pool (Figure 3C).

Taken as a whole our data clearly show that dissimilar nutritional and metabolic states may result in quite similar responses in sulfate uptake, suggesting that multi-signalling pathways may control the expression of the high-affinity sulfate transporters of the roots. Moreover, the fact that the negative relationships between the levels of nutritional signals (sulfate and GSH) and sulfate uptake capacity of the roots, existing in sulfur-starved plants, are not found in the Cd exposed ones, seems to further support this conclusion.

Although it is difficult to indicate unambiguous nutritional signals, we can make some educated guesses on the general structure of the hypothetical signaling pathways involved in the modulation of sulfate uptake by analyzing the expression pattern of the three *BjSultr1;2* forms under Cd exposure and sulfate limitation. Since *BjSultr1;2a* seems to have lost its capacity to respond to Cd stress, but, at the same time, retains its response to sulfate shortage, we can speculate about the existence of at least two distinct signal transduction pathways. The first one modulates root sulfate uptake, ensuring adequate nutrient supply when plants experience lowering in the sulfate concentration of the soil solution, probably through a *cis*-acting sulfur responsive element as previously suggested [19], whilst the second one we postulate is likely to be involved in meeting sulfate uptake with the plant metabolic sulfur demand, which may increase following heavy-metal stress. All the three *BjSultr1;2* forms are controlled by the first pathway as indicated by the analysis of their respective contribution to the increase in the cumulative amount of the *BjSultr1;2* transcripts under sulfur starvation, but only two forms (*BjSultr1;2b/c*) seem to have retained the ancient characteristic to be controlled by the second regulatory pathway. In this context the differential transcriptional behaviors of the three *BjSultr1;2* forms could be explained by hypothesizing the presence of both “cadmium-” and “sulfur-sensitive” regions in the promoter of an ancient *Brassica Sultr1;2* form, which – following polyploidization – may have evolved in sub-functionalized forms, whose combined actions result in molecular and physiological responses to Cd exposure and sulfate limitation similar to those known in species with non-redundant genome [8,9,15]. If this were not the case an interference of the metal with the signal transduction pathways involved in the regulation of sulfate uptake should be postulated, as suggested in the recent paper of Shahbaz and co-workers [51], in which they extensively discuss the effect of copper accumulation on sulfur metabolism-related gene expression. However, copper stress in *Brassica* seems to produce significant increases in both sulfate uptake and tissue sulfate content without substantially altering plant sulfur demand [52], differently from Cd exposure which produces increases in sulfate uptake closely related to the strength of the additional sink for thiols it induces (Additional file 11). Moreover, if any sort of Cd interference occurs we should also suppose the existence of at least two signal transduction pathways controlling the expression of BjSultr1;2a/b/c under Cd exposure: the first inhibited by Cd, and the second Cd insensitive. Finally, we cannot exclude that other molecular mechanisms may be involved in the differential expression of the three *BjSultr1;2* forms under Cd stress, as for instance those suggested for rice phosphate transporters [53]; it could be interesting to investigate if the short 9 bp-insertion located at the 5′ end of the *BjSultr1;2a* coding sequence (Additional file 8) can play a role in the regulation of its expression.

## Conclusions

Taken as a whole our data agree with the main molecular and physiological evidence obtained in Arabidopsis which support the idea that the regulation of AtSultr1;1 and AtSultr1;2 – the two transporters mediating sulfate uptake from the soil solution – must necessarily involve independent signaling pathways, as extensively shown by Rouached and co-workers [15,54]. Moreover, we can also conclude that different sulfur nutritional and metabolic conditions may be perceived by a single sulfate transporter gene. Such a finding reveals that the mechanisms involved in sulfate uptake regulation may be more complex than previously thought, and partially accounts for the lack of unambiguous nutritional signals, since the activity of each transporter may result from a complex interplay among multiple regulatory pathways.

## Abbreviations

Cd: Cadmium; Cys: Cysteine; GSH: Glutathione; HCys: DL-homocysteine; HEPES: 4-(2-HydroxyEthyl)-1-PiperazineEthaneSulfonic acid; ICP-MS: Inductively coupled plasma-mass spectrometry; Met: Methionine; NPT: NonProtein thiol; OAS: *O*-acetylserine; ORF: Open reading frame; PC: Phytochelatin; PCR: Polymerase chain reaction; RT-PCR: Reverse transcription - PCR; SE: Standard error of the mean.

## Competing interests

The authors declare that they have no competing interests.

## Authors’ contributions

CL and FFN conceived and designed the experiments and wrote the manuscript. CL and BG carried out the physiological and molecular analyses. BG, FFN, MC, and JCD conceived and performed the experiments with yeast. GL performed ICP-MS analysis. GAS acquired the funds. CL, JCD, MC, GAS, and FFN discussed and critical revised the manuscript. All authors read and approved the final manuscript.

## Supplementary Material

Additional file 1 **Dendrogram showing sulfate transporter family of *Arabidopsis thaliana *and high-affinity sulfate transporters of *Brassica juncea*.** The dendrogram was constructed on the bases of amino acid sequences using MEGA 5.05 software. Accession numbers for *A. thaliana* (TAIR; http://www.arabidopsis.org/) are: AtSultr1;1, At4g08620; AtSultr1;2, At1g78000; AtSultr1;3, At1g22150; AtSultr2;1, At5g10180; AtSultr2;2, At1g77990; AtSultr3;1, At3g51895; AtSultr3;2, At4g02700; AtSultr3;3, At1g23090; AtSultr3;4, At3g15990; AtSultr3;5, At5g19600; AtSultr4;1, At5g13550; AtSultr4;2, At3g12520. Accession numbers for *B. juncea* (GenBank; http://www.ncbi.nlm.nih.gov/genbank/) are: BjSultr1;1, JX896426; BjSultr1;2a, JX896427; BjSultr1;2b, JX896428; BjSultr1;2c, JX896429. Click here for file

Additional file 2 **Nucleotide identity (%) between the coding sequences of the three *BjSultr1;2 *variants and other *Brassica Sultr1;2 *coding sequences.** Click here for file

Additional file 3 **Dendrogram showing high affinity sulfate transporters of *Arabidopsis thaliana*, *Brassica juncea*, *Brassica napus*, and *Brassica rapa*.** The dendrogram was constructed on the bases of amino acid sequences using MEGA 5.05 software. Accession numbers for *A. thaliana* (TAIR; http://www.arabidopsis.org/) are: AtSultr1;1, At4g08620; AtSultr1;2, At1g78000. Accession numbers for *B. juncea* and *B. napus* (GenBank; http://www.ncbi.nlm.nih.gov/genbank/) are: BjSultr1;1, JX896426; BjSultr1;2a, JX896427; BjSultr1;2b, JX896428; BjSultr1;2c, JX896429; BnSultr1;1, AJ416460; BnSultr1;2, AJ311388. Accession numbers for *B. rapa* (BRAD; http://brassicadb.org/brad/) are: Bra022623; Bra015641; Bra008340. Click here for file

Additional file 4 **Phenotypic complementation of the yeast double sulfate transporter mutant CP154-7A by the sulfate transporters of *Brassica juncea*.** Yeast mutant cells expressing *BjSultr1;1*, *BjSultr1;2a*, *BjSultr1;2b*, and *BjSultr1;2c* under the control of the galactose-inducible GAL10 promoter or harboring the empty pESC-TRP vector were grown at 28°C for 3 d on a minus-sulfur minimal medium (-S) or on minimal media containing 100 μM sulfate (SO_4_ ^2-^) or 100 μM DL-homocysteine (HCys) as sole sulfur sources. Click here for file

Additional file 5 **Growth curves of complemented yeast cells.** (A, B, C, D) Complemented yeasts were incubated at 28°C for 25 h in liquid media containing different sulfate concentrations (● 0 μM; ○ 1 μM; ▼ 2.5 μM; ∆ 5 μM; ■ 7.5 μM; □ 10 μM; ◆ 25 μM; ◇ 50 μM; ▲ 100 μM) or 100 μM HCys (▽) as sole sulfur source. Absorbance was measured at 600 nm (*A*_600_) along time. Data points and error bars are means and SE of two experiments performed in triplicate (*n* = 6). Click here for file

Additional file 6 **Sulfate content in complemented yeast cells.** Complemented yeast cells expressing BjSultr1;1, BjSultr1;2a, BjSultr1;2b, and BjSultr1;2c were incubated in liquid media containing different sulfate concentrations as sole sulfur source. At the end of the incubation period yeasts were harvested and processed to determine their sulfate content. Values are means ± SE of two experiments run in triplicate (*n* = 6). Click here for file

Additional file 7**Growth analysis of CP154-7A yeast mutant expressing an high- or a low-affinity sulfate transporter of *Zea mays *(ZmST1;1) or *Arabidopsis thaliana *(AtSultr2;1), respectively.***ZmST1;1* and *AtSultr2;1* coding sequences were amplified by RT-PCR from total RNA isolated from maize and Arabidopsis roots, respectively, and cloned in the pESC-TRP vector as described in Methods. (A) Complemented yeast cells were incubated in liquid media containing two sulfate concentrations (0.1 or 0.5 mM) as sole sulfur source. Yeast growth was monitored by measuring the *A*_600_ nm at different times. (●) ZmST1;1 at 0.1 mM sulfate; (○) ZmST1;1 at 0.5 mM sulfate; (▼) AtSultr2;1 at 0.1 mM sulfate; (▽) AtSultr2;1 at 0.5 mM sulfate. (B) Growth curves of yeast cells expressing ZmST1;1 (● 0 μM; ○ 1 μM; ▼ 2.5 μM; △ 5 μM; ■ 7.5 μM; □ 10 μM; ◆ 25 μM; ◇ 50 μM; ▲ 100 μM). (C) Growth curves of yeast cells expressing AtSultr2;1 (● 0 μM; ○ 0.05 mM; ▼ 0.1 mM; △ 0.15 mM; ■ 0.2 mM; □ 0.25 mM; ◆ 0.5 mM; ◇ 1 mM; ▲ 1.5 mM; ▽ 2 mM;  2.5 mM;  3 mM). (D, E) Estimation of the growth constant (k_G_) for sulfate. The duplication times (dt) of the complemented yeast cells were calculated by fitting the equation *A*_600_(t) = *A*_600_(t_0_) e^kt^ to the experimental data reported in B and C. k_G_ was determined by expressing the growth rates (dt^-1^) of complemented yeasts as a function of sulfate concentrations in the media, and by fitting the Michaelis-Menten equation to the data. Results reveal that the k_G_ values for sulfate were similar to the k_M_ values measured for each sulfate transporters by using conventional methods (Nocito *et al. Plant Physiol*, 2006 **141:**1138-1148; Takahashi *et al. Plant J*, 2000 **23:**171-182). Data points and error bars are means and SE of two experiments performed in triplicate (*n* = 6).Click here for file

Additional file 8 **Alignment of nucleotide sequences of the three *BjSultr1;2 *forms.** Shared nucleotides are highlighted in grey. Click here for file

Additional file 9 **Changes in the transcript relative amount of the three *BjSultr1;2 *forms in the roots of *Brassica juncea *grown under Cd exposure or sulfate limitation.** Plants were exposed to 25 μM Cd^2+^ for 48 h (+Cd) or grown under 10 μM SO_4_ ^2-^ for 10 days (-S). Control plants were grown under 200 μM SO_4_ ^2-^ and were not exposed to cadmium. The entire ORFs of the three *BjSultr1;2* forms were amplified and PCR products were digested with ClaI endonuclease, electrophoresed on agarose gel, and finally stained with SYBR Green I. Signals were detected using a laser scanner with 532 nm laser and 526 nm filter and densitometrically analyzed using ImageJ 1.46 software. cDNA loading was normalized using *BjTub* as an internal control. Bars and error bars are means and SE of three independent experiments run in triplicate (*n* = 9). Asterisks indicate significant differences between control and treated plants (*P* ≤ 0.001). Click here for file

Additional file 10 **RT-PCR analyses of the three *BjSultr1;2 *forms in the roots of *Brassica juncea *exposed to 10 μM Cd.** Plants were exposed or not to 10 μM Cd^2+^ for 48 h. (A) The entire ORFs of the three *BjSultr1;2* forms were amplified and PCR products were digested with ClaI endonuclease, electrophoresed on agarose gel, and finally stained with SYBR Green I. cDNA loading was normalized using *BjTub* as an internal control. Signals were detected using a laser scanner with 532 nm laser and 526 nm filter. A representative set of data from three independent experiments is given. (B) Densitometric analysis. Arrows indicate the relative position of each electrophoretic band obtained after digestion of PCR products with ClaI. (C) Statistical analysis. Bars and error bars are means and SE of three independent experiments run in triplicate (*n* = 9). Asterisks indicate significant differences between control and treated plants (*P* ≤ 0.001). Click here for file

Additional file 11 **Relationship between NPT content and sulfate uptake capacity in plant of *Brassica juncea *exposed to different Cd concentrations.** Plants were exposed for 48 h to differentCd^2+^ concentrations: 0 (white), 10 (grey), and 25 (black) μM. Circles, roots; triangles, shoots. Data points and error bars are means and SE of three experiments run in triplicate (*n* = 9). Click here for file
