# Supplementary material for: Cadmium exposure and sulfate limitation reveal differences in the transcriptional control of three sulfate transporter (Sultr1;2) genes in Brassica juncea
Source: BMC Plant Biol. 2014 May 16;14:132. doi: 10.1186/1471-2229-14-132 (PMC4049391; doi:10.1186/1471-2229-14-132)

**Additional file 1 Dendrogram showing sulfate transporter family of *Arabidopsis thaliana* and high-affinity sulfate transporters of *Brassica juncea*.** The dendrogram was constructed on the bases of amino acid sequences using MEGA 5.05 software. Accession numbers for *A. thaliana* (TAIR; http://www.arabidopsis.org/) are: AtSultr1;1, At4g08620; AtSultr1;2, At1g78000; AtSultr1;3, At1g22150; AtSultr2;1, At5g10180; AtSultr2;2, At1g77990; AtSultr3;1, At3g51895; AtSultr3;2, At4g02700; AtSultr3;3, At1g23090; AtSultr3;4, At3g15990; AtSultr3;5, At5g19600; AtSultr4;1, At5g13550; AtSultr4;2, At3g12520. Accession numbers for *B. juncea* (GenBank; http://www.ncbi.nlm.nih.gov/genbank/) are: BjSultr1;1, JX896426; BjSultr1;2a, JX896427; BjSultr1;2b, JX896428; BjSultr1;2c, JX896429.

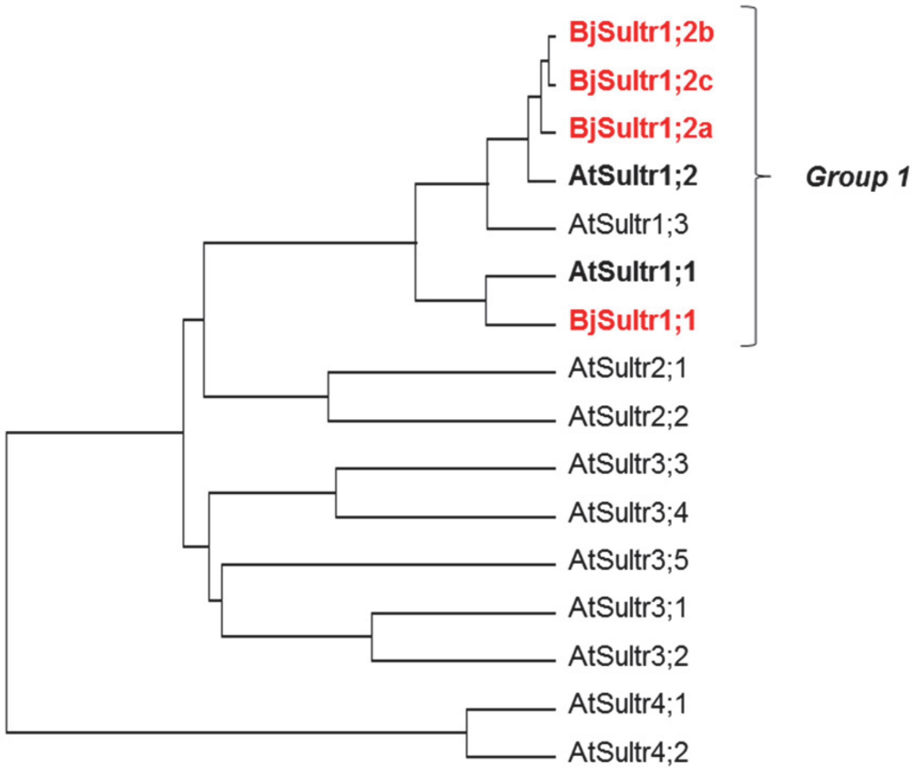

Supplement: Additional file 1 — Dendrogram showing sulfate transporter family of Arabidopsis thaliana and high-affinity sulfate transporters of Brassica juncea. The dendrogram was constructed on the bases of amino acid sequences using MEGA 5.05 software. Accession numbers for A. thaliana (TAIR; http://www.arabidopsis.org/) are: AtSultr1;1, At4g08620; AtSultr1;2, At1g78000; AtSultr1;3, At1g22150; AtSultr2;1, At5g10180; AtSultr2;2, At1g77990; AtSultr3;1, At3g51895; AtSultr3;2, At4g02700; AtSultr3;3, At1g23090; AtSultr3;4, At3g15990; AtSultr3;5, At5g19600; AtSultr4;1, At5g13550; AtSultr4;2, At3g12520. Accession numbers for B. juncea (GenBank; http://www.ncbi.nlm.nih.gov/genbank/) are: BjSultr1;1, JX896426; BjSultr1;2a, JX896427; BjSultr1;2b, JX896428; BjSultr1;2c, JX896429. [file 1471-2229-14-132-S1.pdf]
