# Supplementary material for: Cadmium exposure and sulfate limitation reveal differences in the transcriptional control of three sulfate transporter (Sultr1;2) genes in Brassica juncea
Source: BMC Plant Biol. 2014 May 16;14:132. doi: 10.1186/1471-2229-14-132 (PMC4049391; doi:10.1186/1471-2229-14-132)
Supplement: Additional file 2 — Nucleotide identity (%) between the coding sequences of the three BjSultr1;2 variants and other Brassica Sultr1;2 coding sequences. [file 1471-2229-14-132-S2.docx]

**Additional file 2 Nucleotide identity (%) between the coding sequences of the three *BjSultr1;2* variants and other *Brassica* *Sultr1;2* coding sequences.**

| ***B. juncea***  ***Sultr1;2* clones** | ***Brassica* *Sultr1;2* clones** | | |
| --- | --- | --- | --- |
|  | ***Bra015641*** | ***Bra008340*** | ***BnSultr1;2*** |
|  | **%** | | |
| *BjSultr1;2a* (1968 bp) | 98 | 91 | 95 |
| *BjSultr1;2b* (1959 bp) | 92 | 95 | 92 |
| *BjSultr1;2c* (1959 bp) | 91 | 99 | 94 |
