# Supplementary material for: Cadmium exposure and sulfate limitation reveal differences in the transcriptional control of three sulfate transporter (Sultr1;2) genes in Brassica juncea
Source: BMC Plant Biol. 2014 May 16;14:132. doi: 10.1186/1471-2229-14-132 (PMC4049391; doi:10.1186/1471-2229-14-132)

Additional file 3 Dendrogram showing high affinity sulfate transporters of *Arabidopsis thaliana*, *Brassica juncea*, *Brassica napus*, and *Brassica rapa*. The dendrogram was constructed on the bases of amino acid sequences using MEGA 5.05 software. Accession numbers for *A. thaliana* (TAIR; http://www.arabidopsis.org/) are: AtSultr1;1, At4g08620; AtSultr1;2, At1g78000. Accession numbers for *B. juncea* and *B. napus* (GenBank; http://www.ncbi.nlm.nih.gov/genbank/) are: BjSultr1;1, JX896426; BjSultr1;2a, JX896427; BjSultr1;2b, JX896428; BjSultr1;2c, JX896429; BnSultr1;1, AJ416460; BnSultr1;2, AJ311388. Accession numbers for *B. rapa* (BRAD; http://brassicadb.org/brad/) are: Bra022623; Bra015641; Bra008340.

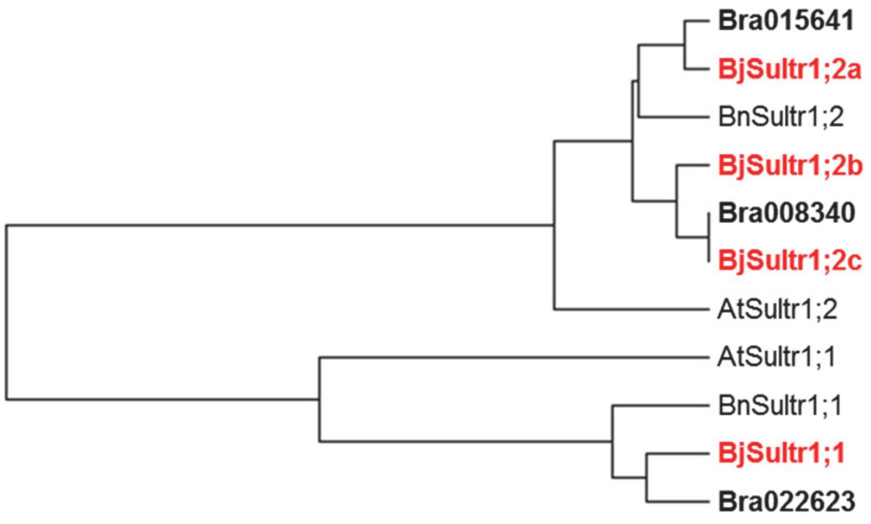

Supplement: Additional file 3 — Dendrogram showing high affinity sulfate transporters of Arabidopsis thaliana, Brassica juncea, Brassica napus, and Brassica rapa. The dendrogram was constructed on the bases of amino acid sequences using MEGA 5.05 software. Accession numbers for A. thaliana (TAIR; http://www.arabidopsis.org/) are: AtSultr1;1, At4g08620; AtSultr1;2, At1g78000. Accession numbers for B. juncea and B. napus (GenBank; http://www.ncbi.nlm.nih.gov/genbank/) are: BjSultr1;1, JX896426; BjSultr1;2a, JX896427; BjSultr1;2b, JX896428; BjSultr1;2c, JX896429; BnSultr1;1, AJ416460; BnSultr1;2, AJ311388. Accession numbers for B. rapa (BRAD; http://brassicadb.org/brad/) are: Bra022623; Bra015641; Bra008340. [file 1471-2229-14-132-S3.pdf]
