# Supplementary material for: Cadmium exposure and sulfate limitation reveal differences in the transcriptional control of three sulfate transporter (Sultr1;2) genes in Brassica juncea
Source: BMC Plant Biol. 2014 May 16;14:132. doi: 10.1186/1471-2229-14-132 (PMC4049391; doi:10.1186/1471-2229-14-132)

Additional file 4 Phenotypic complementation of the yeast double sulfate transporter mutant CP154-7A by the sulfate transporters of *Brassica juncea*. Yeast mutant cells expressing *BjSultr1;1*, *BjSultr1;2a*, *BjSultr1;2b*, and *BjSultr1;2c* under the control of the galactose-inducible GAL10 promoter or harboring the empty pESC-TRP vector were grown at 28°C for 3 d on a minus-sulfur minimal medium (-S) or on minimal media containing 100 μM sulfate (SO_4^{2-}) or 100 μM DL-homocysteine (HCys) as sole sulfur sources.

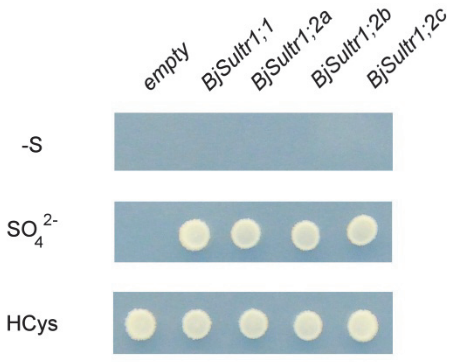

Supplement: Additional file 4 — Phenotypic complementation of the yeast double sulfate transporter mutant CP154-7A by the sulfate transporters of Brassica juncea. Yeast mutant cells expressing BjSultr1;1, BjSultr1;2a, BjSultr1;2b, and BjSultr1;2c under the control of the galactose-inducible GAL10 promoter or harboring the empty pESC-TRP vector were grown at 28°C for 3 d on a minus-sulfur minimal medium (-S) or on minimal media containing 100 μM sulfate (SO4 2-) or 100 μM DL-homocysteine (HCys) as sole sulfur sources. [file 1471-2229-14-132-S4.pdf]
