# Supplementary material for: Cadmium exposure and sulfate limitation reveal differences in the transcriptional control of three sulfate transporter (Sultr1;2) genes in Brassica juncea
Source: BMC Plant Biol. 2014 May 16;14:132. doi: 10.1186/1471-2229-14-132 (PMC4049391; doi:10.1186/1471-2229-14-132)

Additional file 5 Growth curves of complemented yeast cells. (A, B, C, D) Complemented yeasts were incubated at 28°C for 25 h in liquid media containing different sulfate concentrations (● 0 μM; ○ 1 μM; ▼ 2.5 μM; △ 5 μM; ■ 7.5 μM; □ 10 μM; ◆ 25 μM; ◇ 50 μM; ▲ 100 μM) or 100 μM HCys (▽) as sole sulfur source. Absorbance was measured at 600 nm (A_{600}) along time. Data points and error bars are means and SE of two experiments performed in triplicate (n = 6).

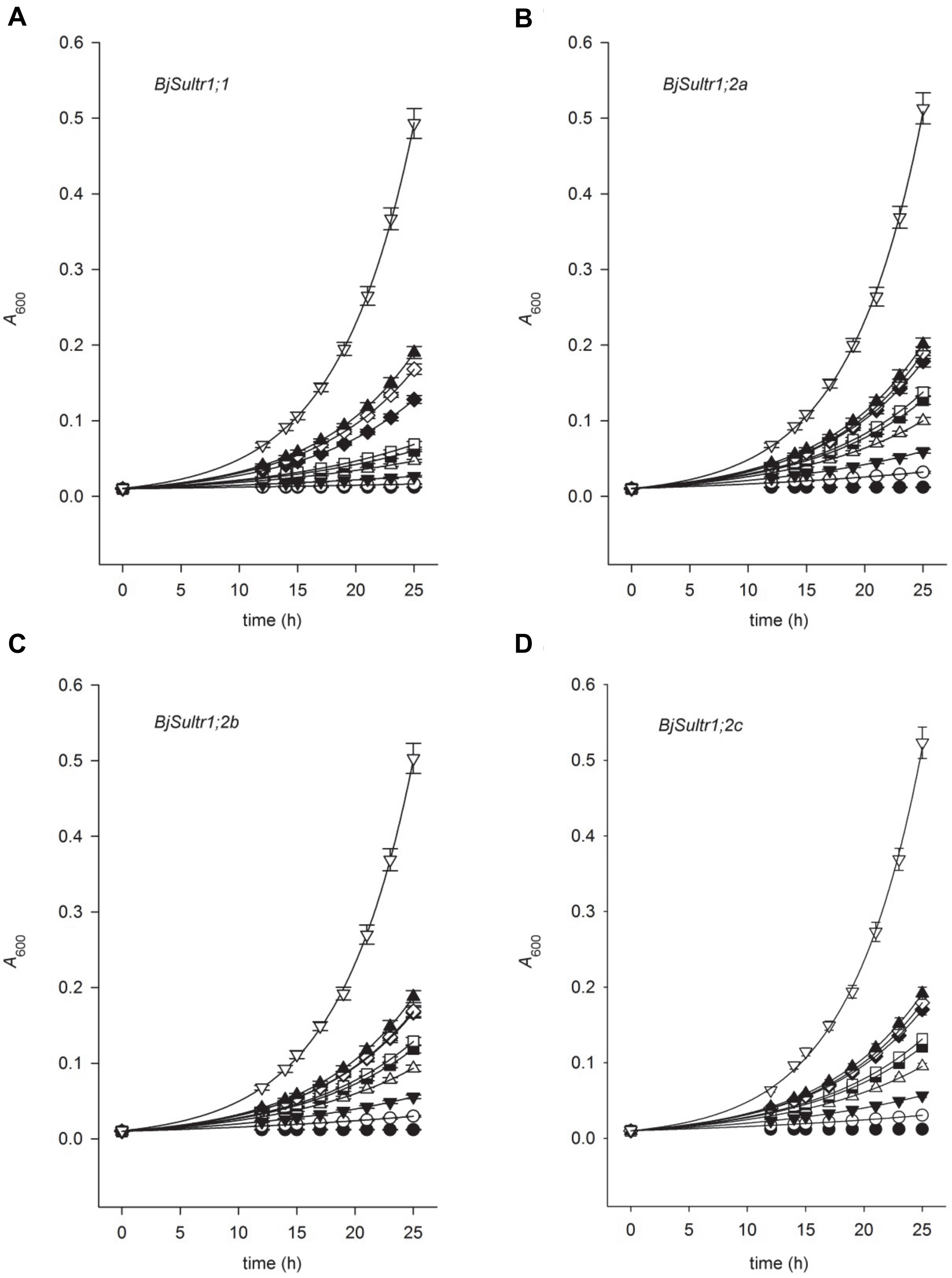

Supplement: Additional file 5 — Growth curves of complemented yeast cells. (A, B, C, D) Complemented yeasts were incubated at 28°C for 25 h in liquid media containing different sulfate concentrations (● 0 μM; ○ 1 μM; ▼ 2.5 μM; ∆ 5 μM; ■ 7.5 μM; □ 10 μM; ◆ 25 μM; ◇ 50 μM; ▲ 100 μM) or 100 μM HCys (▽) as sole sulfur source. Absorbance was measured at 600 nm (A600) along time. Data points and error bars are means and SE of two experiments performed in triplicate (n = 6). [file 1471-2229-14-132-S5.pdf]
