# Supplementary material for: Cadmium exposure and sulfate limitation reveal differences in the transcriptional control of three sulfate transporter (Sultr1;2) genes in Brassica juncea
Source: BMC Plant Biol. 2014 May 16;14:132. doi: 10.1186/1471-2229-14-132 (PMC4049391; doi:10.1186/1471-2229-14-132)
Supplement: Additional file 6 — Sulfate content in complemented yeast cells. Complemented yeast cells expressing BjSultr1;1, BjSultr1;2a, BjSultr1;2b, and BjSultr1;2c were incubated in liquid media containing different sulfate concentrations as sole sulfur source. At the end of the incubation period yeasts were harvested and processed to determine their sulfate content. Values are means ± SE of two experiments run in triplicate (n = 6). [file 1471-2229-14-132-S6.pdf]

Additional file 6 Sulfate content in complemented yeast cells. Complemented yeast cells expressing BjSultr1;1, BjSultr1;2a, BjSultr1;2b, and BjSultr1;2c were incubated in liquid media containing different sulfate concentrations as sole sulfur source. At the end of the incubation period yeasts were harvested and processed to determine their sulfate content. Values are means ± SE of two experiments run in triplicate (*n* = 6).

**[SO_4^{2-}]**	**SO_4^{2-} content**			
	**BjSultr1;1**	**BjSultr1;2a**	**BjSultr1;2b**	**BjSultr1;2c**
**µM**	**nmol A_{600}^{-1}**			
1	2.99 ± 0.13	2.80 ± 0.14	3.01 ± 0.15	2.93 ± 0.07
2.5	2.80 ± 0.11	2.88 ± 0.13	2.94 ± 0.13	2.85 ± 0.12
5	2.95 ± 0.14	2.98 ± 0.14	2.98 ± 0.12	2.93 ± 0.11
7.5	2.87 ± 0.12	3.19 ± 0.13	3.12 ± 0.15	3.13 ± 0.13
10	2.80 ± 0.15	3.02 ± 0.10	3.05 ± 0.14	3.08 ± 0.13
25	2.97 ± 0.12	2.88 ± 0.07	3.17 ± 0.09	2.89 ± 0.12
50	3.13 ± 0.13	2.97 ± 0.13	3.06 ± 0.13	2.75 ± 0.10
100	2.84 ± 0.12	3.01 ± 0.12	2.99 ± 0.13	2.90 ± 0.12
