# Supplementary material for: Cadmium exposure and sulfate limitation reveal differences in the transcriptional control of three sulfate transporter (Sultr1;2) genes in Brassica juncea
Source: BMC Plant Biol. 2014 May 16;14:132. doi: 10.1186/1471-2229-14-132 (PMC4049391; doi:10.1186/1471-2229-14-132)
Supplement: Additional file 7 — Growth analysis of CP154-7A yeast mutant expressing an high- or a low-affinity sulfate transporter of Zea mays (ZmST1;1) or Arabidopsis thaliana (AtSultr2;1), respectively.ZmST1;1 and AtSultr2;1 coding sequences were amplified by RT-PCR from total RNA isolated from maize and Arabidopsis roots, respectively, and cloned in the pESC-TRP vector as described in Methods. (A) Complemented yeast cells were incubated in liquid media containing two sulfate concentrations (0.1 or 0.5 mM) as sole sulfur source. Yeast growth was monitored by measuring the A600 nm at different times. (●) ZmST1;1 at 0.1 mM sulfate; (○) ZmST1;1 at 0.5 mM sulfate; (▼) AtSultr2;1 at 0.1 mM sulfate; (▽) AtSultr2;1 at 0.5 mM sulfate. (B) Growth curves of yeast cells expressing ZmST1;1 (● 0 μM; ○ 1 μM; ▼ 2.5 μM; △ 5 μM; ■ 7.5 μM; □ 10 μM; ◆ 25 μM; ◇ 50 μM; ▲ 100 μM). (C) Growth curves of yeast cells expressing AtSultr2;1 (● 0 μM; ○ 0.05 mM; ▼ 0.1 mM; △ 0.15 mM; ■ 0.2 mM; □ 0.25 mM; ◆ 0.5 mM; ◇ 1 mM; ▲ 1.5 mM; ▽ 2 mM; 2.5 mM; 3 mM). (D, E) Estimation of the growth constant (kG) for sulfate. The duplication times (dt) of the complemented yeast cells were calculated by fitting the equation A600(t) = A600(t0) ekt to the experimental data reported in B and C. kG was determined by expressing the growth rates (dt-1) of complemented yeasts as a function of sulfate concentrations in the media, and by fitting the Michaelis-Menten equation to the data. Results reveal that the kG values for sulfate were similar to the kM values measured for each sulfate transporters by using conventional methods (Nocito et al. Plant Physiol, 2006 141:1138-1148; Takahashi et al. Plant J, 2000 23:171-182). Data points and error bars are means and SE of two experiments performed in triplicate (n = 6). [file 1471-2229-14-132-S7.pdf]

Additional file 7 Growth analysis of CP154-7A yeast mutant expressing an high- or a low-affinity sulfate transporter of *Zea mays* (ZmST1;1) or *Arabidopsis thaliana* (AtSultr2;1), respectively. *ZmST1;1* and *AtSultr2;1* coding sequences were amplified by RT-PCR from total RNA isolated from maize and Arabidopsis roots, respectively, and cloned in the pESC-TRP vector as described in Methods. (A) Complemented yeast cells were incubated in liquid media containing two sulfate concentrations (0.1 or 0.5 mM) as sole sulfur source. Yeast growth was monitored by measuring the A_{600} nm at different times. The growth rates (expressed as reciprocals of the duplication times, dt^{-1}) of the yeast cells expressing the high-affinity sulfate transporter ZmST1;1 were 0.102 ± 0.002 h^{-1} and 0.103 ± 0.002 h^{-1} at 0.1 (●) and 0.5 (○) mM sulfate external concentration, respectively. In the same media yeast cells expressing the low-affinity sulfate transporter AtSultr2;1 showed growth rates significantly lower, which increased from 0.021 ± 0.006 $h^{-1}$to 0.063 ± 0.001 h^{-1} moving the external sulfate concentration from 0.1 (▼) to 0.5 (▽) mM. (B) Growth curves of yeast cells expressing ZmST1;1 (● 0 μM; ○ 1 μM; ▼ 2.5 μM; △ 5 μM; ■ 7.5 μM; □ 10 μM; ◆ 25 μM; ◇ 50 μM; ▲ 100 μM). (C) Growth curves of yeast cells expressing AtSultr2;1 (● 0 μM; ○ 0.05 mM; ▼ 0.1 mM; △ 0.15 mM; ■ 0.2 mM; □ 0.25 mM; ◆ 0.5 mM; ◇ 1 mM; ▲ 1.5 mM; ▽ 2 mM; ⬢ 2.5 mM; ⬡ 3 mM). (D, E) Estimation of the growth constant (k_G) for sulfate. The duplication times (dt) of the complemented yeast cells were calculated by fitting the equation $A_{600}(t) = A_{600}(t_0)\ e^{kt}$ to the experimental data reported in B and C. k_G was determined by expressing the growth rates (dt^{-1}) of complemented yeasts as a function of sulfate concentrations in the media, and by fitting the Michaelis-Menten equation to the data. Results reveal that the k_G values for sulfate were similar to the k_M values measured for each sulfate transporters by using conventional methods (Nocito *et al. Plant Physiol*, 2006 **141:**1138-1148; Takahashi *et al. Plant J*, 2000 **23:**171-182). Data points and error bars are means and SE of two experiments performed in triplicate (n = 6).

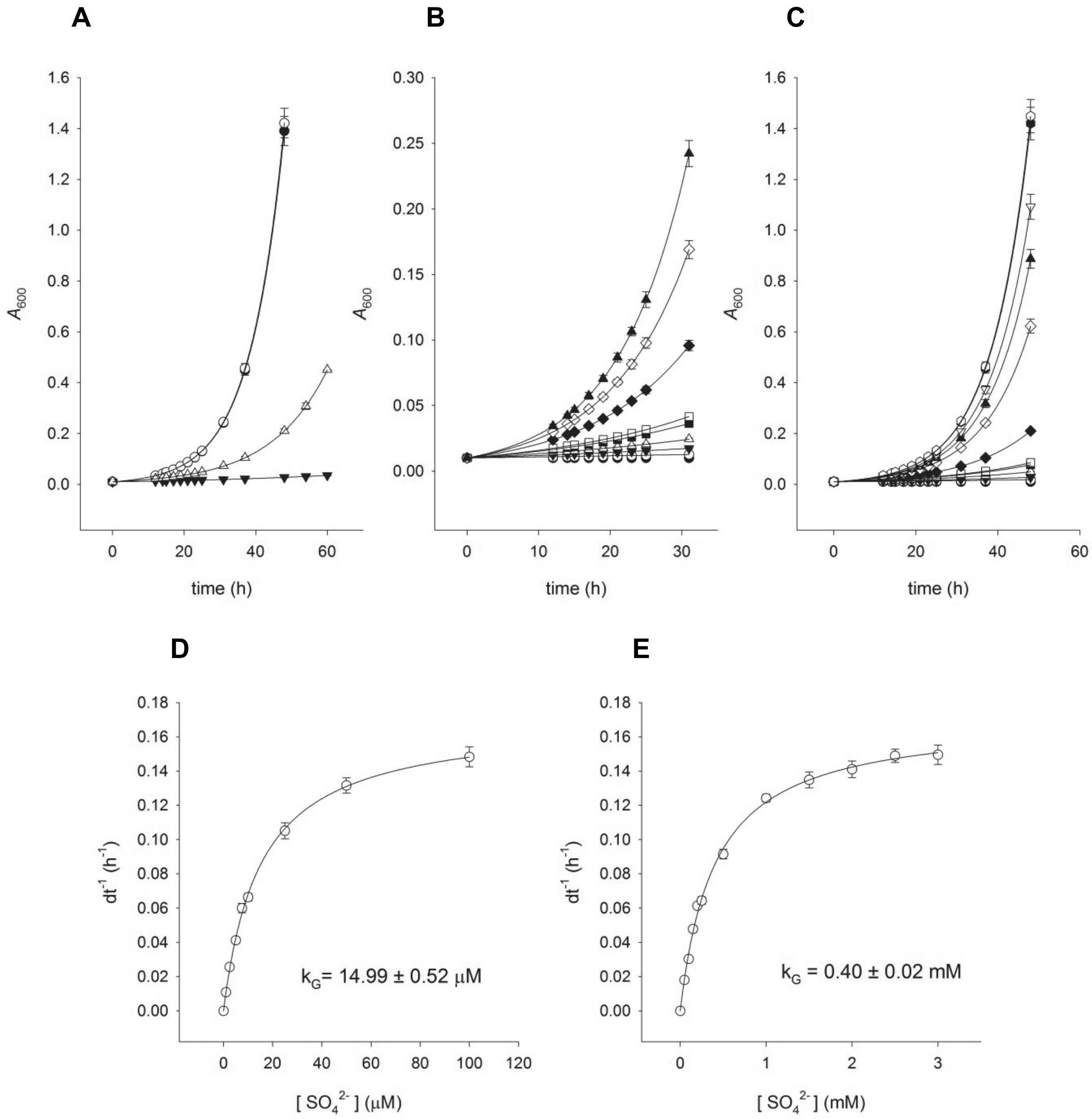

A
B
C
A_{600}
time (h)
D
E
dt^{-1} (h^{-1})
k_G= 14.99 ± 0.52 μM
k_G = 0.40 ± 0.02 mM
[SO_4^{2-}] (μM)
[SO_4^{2-}] (mM)
