# Supplementary material for: Cadmium exposure and sulfate limitation reveal differences in the transcriptional control of three sulfate transporter (Sultr1;2) genes in Brassica juncea
Source: BMC Plant Biol. 2014 May 16;14:132. doi: 10.1186/1471-2229-14-132 (PMC4049391; doi:10.1186/1471-2229-14-132)
Supplement: Additional file 8 — Alignment of nucleotide sequences of the three BjSultr1;2 forms. Shared nucleotides are highlighted in grey. [file 1471-2229-14-132-S8.pdf]

Additional file 8 Alignment of nucleotide sequences of the three *BjSultr1;2* forms. Shared nucleotides are highlighted in grey.

```
BjSultr1;2a     1 ATGTCTGGGAGAGCTCATCCTGTGGACGGTGATGTAAGTCCAGCAACGGACGGTGGAGATCTCCCGATCAAATCGTCGCCTCACCGCCATAAAGTTGGGG
BjSultr1;2b     1 ATGTCTGGGAGAGCTCATCCTG---------ATGTAAATCCGGTGACGGATGGTGGAGATCTTCCTATCAAATCGTCGCCTCACCGGCATAAAGTTGGGG
BjSultr1;2c     1 ATGTCTGGGAGAGCTCATCCTG---------ATGTGAATCCTGTGACGGATGGTGGAGATCTTCCGATCAAATCGTCGCCTCACCGGCATAAAGTTGGTG

BjSultr1;2a   101 TCCCACCGAAGCAGAACATGTTCCATGACTTCATGTACACGTTCAAAGAAACTTTCTTCCATGATGATCCTCTCAGAGATTTTAAGGATCAGCCTAAGTC
BjSultr1;2b    92 TCCCACCGAAGCAAAACATGTTCCATGATTTCATGTACACGTTCAAAGAAACCTTCTTTCATGATGATCCTCTCAGACATTTTAAGGATCAGCCTAAGTC
BjSultr1;2c    92 TCCCACCGAAGCAAAACATGTTCCATGATTTCATGTACACGTTCAAAGAAACGTTCTTCCATGATGATCCTCTCAGACACTTTAAGGATCAGCCTAAGTC

BjSultr1;2a   201 CAAACAGTTCATGCTCGGTCTCCAGTCTGTCTTCCCGGTTTTCGATTGGGGACGTAGCTACAATCTCAAGAAGTTTCGTGGTGATCTAATTGCCGGTTTA
BjSultr1;2b   192 CAAAAAGTTCATGCTCGGTCTTCAATCTGTCTTCCCGGTTTTCGATTGGGGACGTAACTATAATCTCAAGAAGTTCCGTGGTGATCTCATTGCCGGTTTA
BjSultr1;2c   192 TAAGAAGTTCATGCTCGGTCTCCAATCTGTGTTCCCGGTTTTCGATTGGGGACGTAACTATAATCTCAAGAAGTTCCGTGGTGATCTCATTGCCGGTTTA

BjSultr1;2a   301 ACTATTGCCAGTCTCTGTATTCCTCAGGATATTGGATACGCTAAGCTCGCTAATTTGGATCCTAAATACGGTTTATATTCGAGTTTTGTACCTCCATTGG
BjSultr1;2b   292 ACTATTGCCAGTCTTTGTATTCCTCAGGATATTGGATATGCTAAGCTTGCAAATCTGGACCCTAAATACGGTTTATATTCGAGTTTTGTTCCACCGTTGG
BjSultr1;2c   292 ACTATTGCCAGTCTTTGTATCCCTCAGGATATTGGATATGCTAAGCTTGCGAACCTGGATCCTAAATACGGTTTATATTCGAGTTTTGTTCCACCGTTGG

BjSultr1;2a   401 TGTATGCATGTATGGGGAGTTCTAGGGACATAGCAATAGGACCCGTCGCTGTGGTTTCTCTGCTGCTAGGTACTCTCCTTCAAGCTGAGGTCGACCCAGT
BjSultr1;2b   392 TGTATGCTTGTATGGGGAGTTCTAGGGATATAGCAATCGGACCTGTCGCTGTGGTTTCTCTTCTGCTAGGTACTCTCCTTCGAGCTGAGATTGATCCAAG
BjSultr1;2c   392 TGTATGCTTGTATGGGGAGTTCTAGGGATATAGCAATTGGACCTGTTGCTGTGGTATCTCTTCTGCTAGGTACTCTCCTTCGAGCTGAGGTTGATCCAAA

BjSultr1;2a   501 CACAAACCCTGATGAGTACCTCCGACTTGCCTTCACCGCCACGTTTTTTGCCGGTGTCACTGAAGCAGCCCTTGGATTCTTTAGGTTAGGGTTCTTGATT
BjSultr1;2b   492 CACAAATCCTGATGAGTATCTCCGACTTGCCTTCACCGCCACGTTTTTCGCTGGTGTCACTGAAGCAGCCCTTGGCTTCTTCAGATTAGGATTCTTGATT
BjSultr1;2c   492 CACAAATCCTGATGAGTATCTCCGACTTGCCTTCACGGCTACGTTTTTTGCCGGTGTCACTGAAGCAGCCCTTGGCTTCTTCAGATTAGGGTTCTTGATT

BjSultr1;2a   601 GATTTCCTTTCACACGCGGCTGTGGTTGGATTCATGGGTGGTGCAGCCATCACAATAGCTCTTCAGCAGCTCAAAGGGTTCCTAGGGATCAAAAAATTCA
BjSultr1;2b   592 GATTTCCTTTCCCATGCGGCTGTGGTTGGATTCATGGGTGGTGCAGCCATCACAATCGCTCTTCAGCAGCTCAAAGGTTTCCTAGGGATCAAAAAGTTCA
BjSultr1;2c   592 GATTTCCTTTCCCATGCGGCTGTGGTTGGATTCATGGGTGGTGCAGCCATCACAATAGCTCTTCAGCAGCTCAAGGGTTTCTTAGGGATCAAGAAATTCA

BjSultr1;2a   701 CCAAGAAAACCGATATCATTGCTGTTTTAGATTCTGTCTTCAGCGCAGCTCATCATGGTTGGAATTGGCAGACTATACTCATTGGTGCATCCTTCTTGAC
BjSultr1;2b   692 CCAAGAAAACTGATATCATCGCTGTTCTAGATTCAGTCTTTAGTGCAGCTCATCATGGCTGGAACTGGCAGACTATACTCATTGGTGCATCCTTCTTGAC
BjSultr1;2c   692 CCAAGAAAACTGATATCATCGCTGTTTTAGATTCAGTCTTTAGCGCAGCTCATCATGGTTGGAATTGGCAGACTATACTCATTGGTGCATCCTTCTTGAC

BjSultr1;2a   801 CTTCCTTCTCACTTCTAAACTCATTGGGAAAAAGAACAAGAAACTGTTCTGGGTTCCGGCGGTTGCACCGTTGATATCAGTTATCATTTCCACCTTCTTT
BjSultr1;2b   792 CTTCCTTCTCACATCTAAGTTCATTGGCAAAAAGAGCAAGAAACTGTTCTGGATTCCAGCGGTTGCACCACTGATATCAGTTATCATTTCCACCTTCTTT
BjSultr1;2c   792 ATTCCTTCTCACATCTAAGTTCATTGGCAAAAAGAGCAAGAAACTGTTCTGGATTCCAGCTGTTGCGCCATTGATATCAGTTATCATCTCCACATTCTTT

BjSultr1;2a   901 GTCTACATAACCCGAGCCGACAAACAAGGAGTCCAAATTGTGAAACATCTTGACAAAGGGATCAATCCTTCCTCTTTTGATCAAATTTACTTCTCCGGAG
BjSultr1;2b   892 GTTTACATAACCAGAGCAGACAAACAAGGAGTCCAAATCGTGAAACACCTTGACAAGGGCATCAACCCTTCATCTTTTAATCAAATATACTTCTCCGGCC
BjSultr1;2c   892 GTCTACATAACCAGAGCTGACAAACAAGGAGTCCAAATCGTGAAACACCTTGACAAAGGCATCAATCCTTCATCGTTCCATCAAATCTACTTCTCCGGAC
```

```
BjSultr1;2a  1001 ATAACTTGGCAAAGGGGATTCGCATCGGTGTTGTCGCTGGAATGGTCGCCCTAACAGAAGCTGTAGCGATTGGAAGAACGTTTGCTGCAATGAAAGACTA
BjSultr1;2b   992 ATTACCTAGCTAAGGGCATTCGCATCGGTGTAGTCGCTGGAATGGTTGCCTTAACGGAAGCTGTGGCTATTGGAAGAACATTTGCTGCAATGAAAGACTA
BjSultr1;2c   992 ATTACCTTGCTAAGGGCATTCGTATCGGTGTAGTCGCTGGAATGGTCGCCTTAACGGAAGCTGTGGCCATTGGAAGAACGTTTGCTGCAATGAAAGACTA

BjSultr1;2a  1101 TCAAATCGACGGTAACAAAGAGATGGTAGCACTAGGTGTTATGAACGTTGTTGGATCAATGTCTTCTTGCTACGTGGCGACAGGATCGTTCTCAAGATCA
BjSultr1;2b  1092 TCAAATAGATGGTAACAAAGAGATGGTAGCACTAGGTGTTATGAACGTTGTTGGGTCCATGTCTTCTTGCTACGTAGCCACTGGATCGTTCTCGAGATCA
BjSultr1;2c  1092 TCAAATCGATGGTAACAAAGAGATGGTAGCACTAGGTGTTATGAACGTTGTTGGTTCCATGTCTTCTTGCTACGTAGCGACTGGATCATTCTCAAGATCA

BjSultr1;2a  1201 GCTGTAAACTTCATGGCTGGATGTCAAACTGCTGTTTCCAACATCATAATGTCAATCGTTGTTCTCTTGACACTGCTCTTCCTCACTCCTCTTTTCAAGT
BjSultr1;2b  1192 GCTGTAAACTTCATGGCTGGATGTCAAACTGCGGTTTCAAACATCATAATGTCAATCGTTGTTCTCTTGACATTGCTTTTCCTCACTCCACTATTTAAGT
BjSultr1;2c  1192 GCTGTAAACTTCATGGCTGGATGTCAAACTGCGGTTTCAAACATCATAATGTCAATCGTTGTTCTCTTGACATTGCTCTTCCTCACTCCGTTGTTTAAGT

BjSultr1;2a  1301 ACACACCAAACGCCATCCTAGCAGCTATTATCATCAACGCAGTGATTCCTCTGATTGATATCCAAGCAGCTATTTTGATCTTCAAGGTCGATAAGCTCGA
BjSultr1;2b  1292 ACACACCAAACGCAATCCTCGCAGCTATTATCATCAATGCTGTGATTCCTTTGATTGATATCCAAGCTGCAATATTGATCTTCAAGGTCGATAAGCTTGA
BjSultr1;2c  1292 ACACACCAAACGCCATCCTCGCAGCTATTATCATCAATGCTGTGATTCCTTTGATTGATATCCAAGCTGCGATATTGATCTTCAAGGTCGATAAGCTTGA

BjSultr1;2a  1401 CTTCATCGCTTGTATGGGAGCTTTCTTTGGCGTCATCTTTGTTTCCGTTGAGATCGGACTTCTTATTGCCGTCTCTATATCGTTTGCTAAGATTCTCTTG
BjSultr1;2b  1392 TTTCGTTGCTTGTATGGGAGCTTTCTTTGGCGTCATCTTTGTTTCTGTTGAGATAGGGCTTCTCATTGCCGTTTCTATATCATTTGCAAAGATTCTCTTG
BjSultr1;2c  1392 CTTCGTTGCTTGTATGGGAGCTTTCTTTGGCGTCATCTTTGCTTCCGTTGAGATAGGGCTTCTCATTGCCGTCTCTATATCATTCGCTAAGATTCTCTTG

BjSultr1;2a  1501 CAAGTAACAAGACCTAGAACCGCAGTTCTTGGAAACATCCCAAGAACTTCAGTATACCGGAACATCCAACAGTATCCTGAAGCCACCATGGTTCCAGGGG
BjSultr1;2b  1492 CAAGTAACAAGACCTAGAACCGCGGTTCTTGGAAACATTCCGAGAACTTCAGTTTATAGAAACATCCAACAGTATCCTGAAGCCACTATGGTTCCAGGGG
BjSultr1;2c  1492 CAAGTAACAAGACCTAGAACGGCGGTTCTTGGAAACATTCCGAGAACTTCAGTTTACAGAAACATCCAACAGTATCCTGAAGCCACTATGGTTCCAGGGG

BjSultr1;2a  1601 TTCTTACCATTCGTGTTGACTCGGCGATTTACTTCTCCAACTCAAATTACGTCAGAGAAAGGATCCAAAGATGGTTACTAGAGGAAGAAGAGAAGGTGAA
BjSultr1;2b  1592 TTCTTACCATTCGTGTTGACTCCGCGATTTACTTCTCCAACTCAAATTACGTCAGAGAAAGGATCCAAAGATGGTTACTAGAGGAAGAAGAGAAAGTGAA
BjSultr1;2c  1592 TTCTCACGATTCGTGTTGACTCAGCCATTTACTTCTCCAACTCAAATTACGTCAGAGAAAGGATCCAAAGATGGTTACTAGAGGAAGAAGAGAAGGTGAA

BjSultr1;2a  1701 AGCAGCAAGCCTACCTAGGATCCAGTTTCTGATCATTGAAATGTCACCTGTTACGGACATCGACACAAGTGGTATTCACGCCCTAGAAGACTTATACAAA
BjSultr1;2b  1692 AGCAGCAAGCCTACCTAGGATCCAGTTTCTAATCATCGAAATGTCACCTGTTACGGACATCGATACAAGTGGTATCCACGCCCTAGAAGACTTATATAAG
BjSultr1;2c  1692 AGCAGCAAGCTTACCTAGGATCCAGTTTCTAATCATCGAGATGTCACCCGTGACGGATATCGATACAAGTGGTATCCACGCCCTAGAAGACTTATACAAA

BjSultr1;2a  1801 TCTCTCCAGAAAAGAGATATTCAGTTGGTTCTTGCGAACCCTGGACCGTTGGTGATTGGCAAGCTACACTTGTCGCACTTTGCTGACATGCTAGGACACG
BjSultr1;2b  1792 TCTCTACAGAAGAGAGATATTCAGCTGATTCTTGCTAATCCTGGACCGTTGGTAATAGGCAAGCTACACTTGTCGCACTTTGCTGATATGTTAGGACAAG
BjSultr1;2c  1792 TCTCTCCAGAAGAGAGATATTCAGCTGATTCTTGCAAATCCTGGACCATTGGTGATAGGCAAATTACACTTGTCTCACTTTGCTGACATGTTGGGATATG

BjSultr1;2a  1901 ACCATATCTTTCTGACGGTGGCTGATGCCGTGGAAGCTTGCTGTCCAAAACTCTCCGACGAGGTCTGA 1968
BjSultr1;2b  1892 ACAATATATTTCTGACGGTGGCTGATGCCGTCGAATCTTGCTGTCCAAAACTCTCCGACGAGGTCTGA 1959
BjSultr1;2c  1892 ACCATATCTTTCTGACGGTGGCTGACGCCGTCGAAGCTTGTGGTCCAAAACTCTCCGACGAGGTCTGA 1959
```
