# Supplementary material for: Cadmium exposure and sulfate limitation reveal differences in the transcriptional control of three sulfate transporter (Sultr1;2) genes in Brassica juncea
Source: BMC Plant Biol. 2014 May 16;14:132. doi: 10.1186/1471-2229-14-132 (PMC4049391; doi:10.1186/1471-2229-14-132)

Additional file 9 Changes in the transcript relative amount of the three *BjSultr1;2* forms in the roots of *Brassica juncea* grown under Cd exposure or sulfate limitation. Plants were exposed to 25 µM Cd^{2+} for 48 h (+Cd) or grown under 10 µM SO_4^{2-} for 10 days (-S). Control plants were grown under 200 µM SO_4^{2-} and were not exposed to cadmium. The entire ORFs of the three *BjSultr1;2* forms were amplified and PCR products were digested with ClaI endonuclease, electrophoresed on agarose gel, and finally stained with SYBR Green I. Signals were detected using a laser scanner with 532 nm laser and 526 nm filter and densitometrically analyzed using ImageJ 1.46 software. cDNA loading was normalized using *BjTub* as an internal control. Bars and error bars are means and SE of three independent experiments run in triplicate (n = 9). Asterisks indicate significant differences between control and treated plants ($P \leq 0.001$).

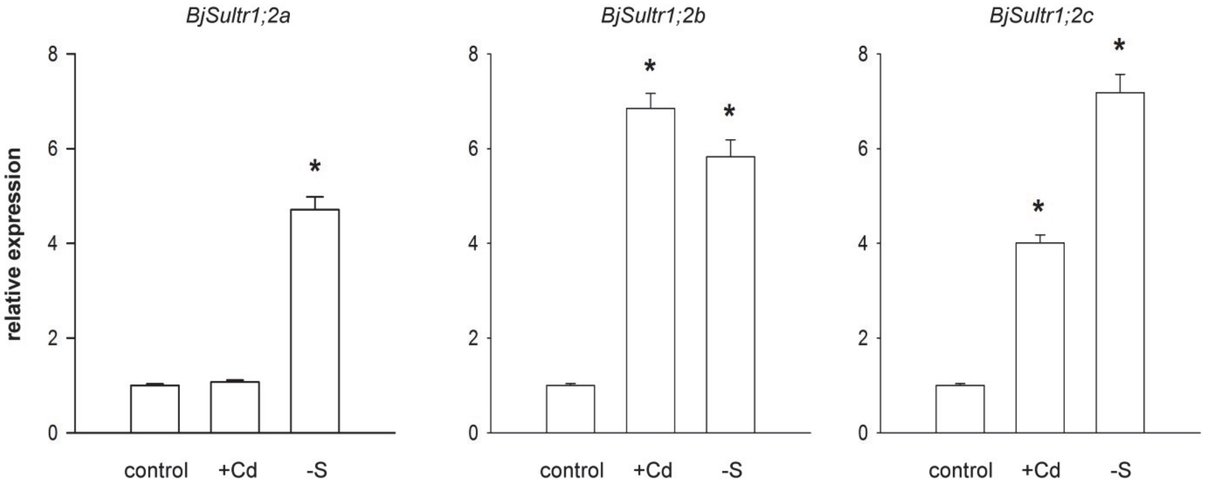

Supplement: Additional file 9 — Changes in the transcript relative amount of the three BjSultr1;2 forms in the roots of Brassica juncea grown under Cd exposure or sulfate limitation. Plants were exposed to 25 μM Cd2+ for 48 h (+Cd) or grown under 10 μM SO4 2- for 10 days (-S). Control plants were grown under 200 μM SO4 2- and were not exposed to cadmium. The entire ORFs of the three BjSultr1;2 forms were amplified and PCR products were digested with ClaI endonuclease, electrophoresed on agarose gel, and finally stained with SYBR Green I. Signals were detected using a laser scanner with 532 nm laser and 526 nm filter and densitometrically analyzed using ImageJ 1.46 software. cDNA loading was normalized using BjTub as an internal control. Bars and error bars are means and SE of three independent experiments run in triplicate (n = 9). Asterisks indicate significant differences between control and treated plants (P ≤ 0.001). [file 1471-2229-14-132-S9.pdf]
