# Supplementary material for: Cadmium exposure and sulfate limitation reveal differences in the transcriptional control of three sulfate transporter (Sultr1;2) genes in Brassica juncea
Source: BMC Plant Biol. 2014 May 16;14:132. doi: 10.1186/1471-2229-14-132 (PMC4049391; doi:10.1186/1471-2229-14-132)
Supplement: Additional file 10 — RT-PCR analyses of the three BjSultr1;2 forms in the roots of Brassica juncea exposed to 10 μM Cd. Plants were exposed or not to 10 μM Cd2+ for 48 h. (A) The entire ORFs of the three BjSultr1;2 forms were amplified and PCR products were digested with ClaI endonuclease, electrophoresed on agarose gel, and finally stained with SYBR Green I. cDNA loading was normalized using BjTub as an internal control. Signals were detected using a laser scanner with 532 nm laser and 526 nm filter. A representative set of data from three independent experiments is given. (B) Densitometric analysis. Arrows indicate the relative position of each electrophoretic band obtained after digestion of PCR products with ClaI. (C) Statistical analysis. Bars and error bars are means and SE of three independent experiments run in triplicate (n = 9). Asterisks indicate significant differences between control and treated plants (P ≤ 0.001). [file 1471-2229-14-132-S10.pdf]

Additional file 10 RT-PCR analyses of the three *BjSultr1;2* forms in the roots of *Brassica juncea* exposed to 10 µM Cd. Plants were exposed or not to 10 µM Cd^{2+} for 48 h. (A) The entire ORFs of the three *BjSultr1;2* forms were amplified and PCR products were digested with ClaI endonuclease, electrophoresed on agarose gel, and finally stained with SYBR Green I. cDNA loading was normalized using *BjTub* as an internal control. Signals were detected using a laser scanner with 532 nm laser and 526 nm filter. A representative set of data from three independent experiments is given. (B) Densitometric analysis. Arrows indicate the relative position of each electrophoretic band obtained after digestion of PCR products with ClaI. (C) Statistical analysis. Bars and error bars are means and SE of three independent experiments run in triplicate (n = 9). Asterisks indicate significant differences between control and treated plants ($P \leq 0.001$).

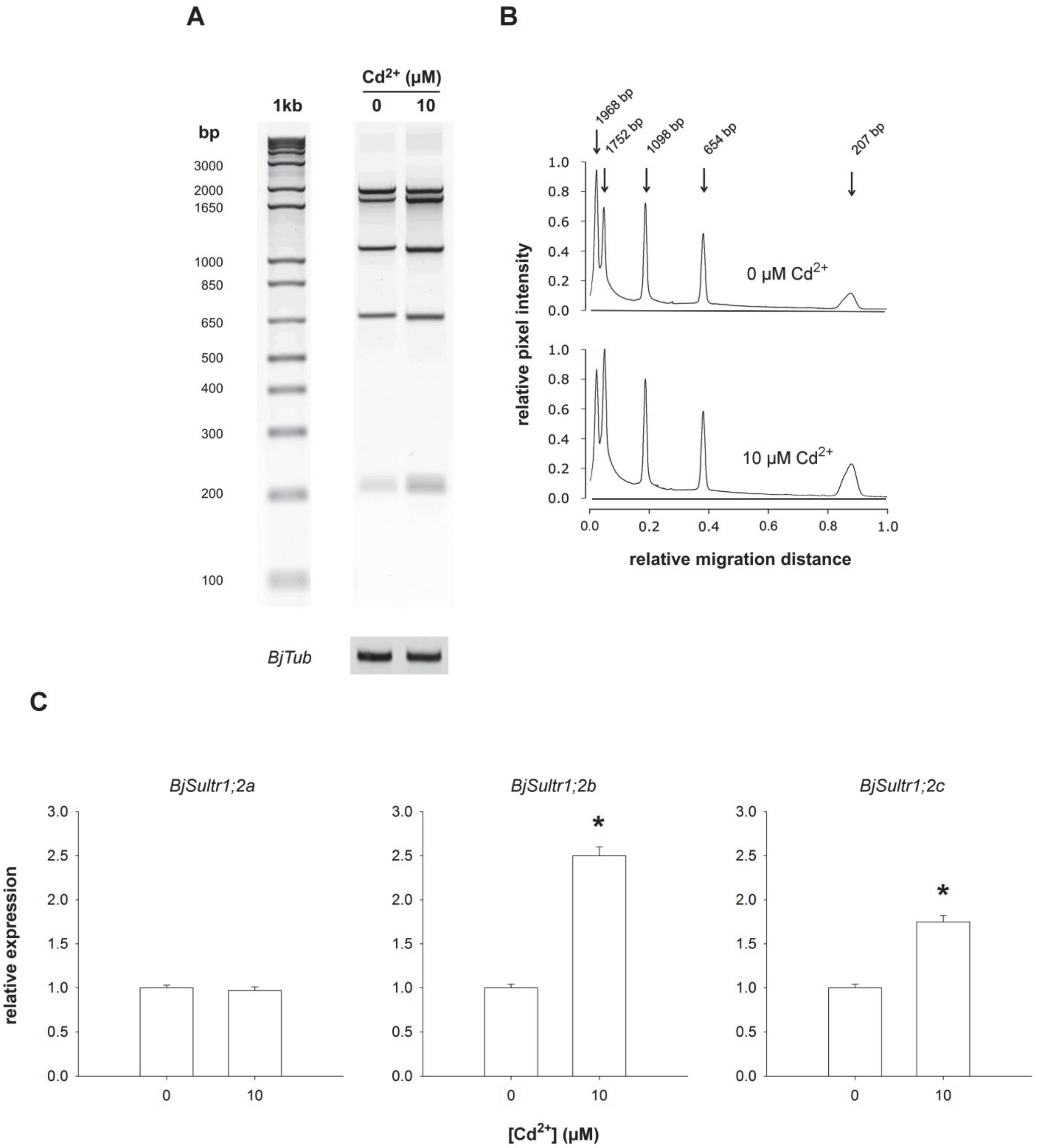

A
B
Cd2+ (μM)
1kb
0
10
bp
3000
2000
1650
1000
850
650
500
400
300
200
100
BjTub
1968 bp
1752 bp
1098 bp
654 bp
207 bp
relative pixel intensity
0 μM Cd2+
10 μM Cd2+
relative migration distance
C
BjSultr1;2a
BjSultr1;2b
BjSultr1;2c
relative expression
[Cd2+] (μM)
