# Supplementary material for: Cadmium exposure and sulfate limitation reveal differences in the transcriptional control of three sulfate transporter (Sultr1;2) genes in Brassica juncea
Source: BMC Plant Biol. 2014 May 16;14:132. doi: 10.1186/1471-2229-14-132 (PMC4049391; doi:10.1186/1471-2229-14-132)

Additional file 11 Relationship between NPT content and sulfate uptake capacity in plant of *Brassica juncea* exposed to different Cd concentrations. Plants were exposed for 48 h to differentCd^{2+} concentrations: 0 (white), 10 (grey), and 25 (black) µM. Circles, roots; triangles, shoots. Data points and error bars are means and SE of three experiments run in triplicate (n = 9).

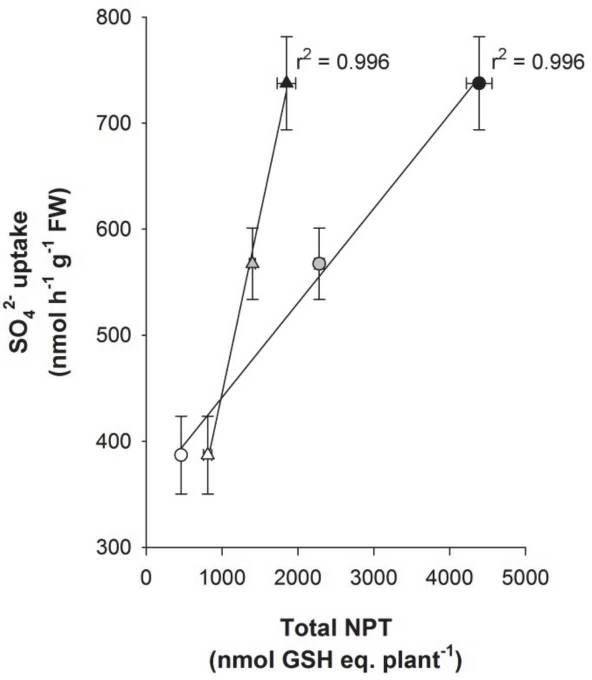

Supplement: Additional file 11 — Relationship between NPT content and sulfate uptake capacity in plant of Brassica juncea exposed to different Cd concentrations. Plants were exposed for 48 h to differentCd2+ concentrations: 0 (white), 10 (grey), and 25 (black) μM. Circles, roots; triangles, shoots. Data points and error bars are means and SE of three experiments run in triplicate (n = 9). [file 1471-2229-14-132-S11.pdf]
